# Evidence on bringing specialised care to the primary level—effects on the Quadruple Aim and cost-effectiveness: a systematic review

**DOI:** 10.1186/s12913-023-10159-6

**Published:** 2024-01-02

**Authors:** Maria Lovén, Laura J. Pitkänen, Markus Paananen, Paulus Torkki

**Affiliations:** 1 Department of Public Health, University of Helsinki, Helsinki, Finland; 2 Mehiläinen Länsi-Pohja, Mehiläinen, Helsinki, Finland; 3 Social and Health Care Services, Western Uusimaa Wellbeing Services County, University of Oulu, Oulu, Finland

**Keywords:** Quadruple Aim, Outreach, Vertical integration, Specialised care, Primary care, Cost-effectiveness, Patient-reported outcomes, Patient experience, Professional satisfaction

## Abstract

**Background:**

To achieve the Quadruple Aim of improving population health, enhancing the patient experience of care, reducing costs and improving professional satisfaction requires reorganisation of health care. One way to accomplish this aim is by integrating healthcare services on different levels. This systematic review aims to determine whether it is cost-effective to bring a hospital specialist into primary care from the perspectives of commissioners, patients and professionals.

**Methods:**

The review follows the PRISMA guidelines. We searched PubMed, Scopus and EBSCO (CINAHL and Academic Search Ultimate) for the period of 1992–2022. In total, 4254 articles were found, and 21 original articles that reported on both quality and costs, were included. The JBI and ROBINS-I tools were used for quality appraisal. In data synthesis, vote counting and effect direction plots were used together with a sign test. The strength of evidence was evaluated with the GRADE.

**Results:**

Cost-effectiveness was only measured in two studies, and it remains unclear. Costs and cost drivers for commissioners were lower in the intervention in 52% of the studies; this proportion rose to 67% of the studies when cost for patients was also considered, while health outcomes, patient experience and professional satisfaction mostly improved but at least remained the same. Costs for the patient, where measured, were mainly lower in the intervention group. Professional satisfaction was reported in 48% of the studies; in 80% it was higher in the intervention group. In 24% of the studies, higher monetary costs were reported for commissioners, whereas the clinical outcomes, patient experience and costs for the patient mainly improved.

**Conclusions:**

The cost-effectiveness of the hospital specialist in primary care model remains inconclusive. Only a few studies have comprehensively calculated costs, evaluating cost drivers. However, it seems that when the service is well organised and the population is large enough, the concept can be profitable for the commissioner also. From the patient’s perspective, the model is superior and could even promote equity through improved access. Professional satisfaction is mostly higher compared to the traditional model. The certainty of evidence is very low for cost and low for quality.

**Trial registration:**

PROSPERO CRD42022325232, 12.4.2022.

**Supplementary Information:**

The online version contains supplementary material available at 10.1186/s12913-023-10159-6.

## Background

Globally, healthcare spending is on the rise; spending on health care more than doubled in real terms between 2000 and 2019, reaching 9.8% of the global gross domestic product [1]. To limit the rise of total costs, it is necessary to seek ways to improve the cost-effectiveness of care. This could involve a better service structure that includes the interfaces of the different care levels. Currently, resources are being wasted because of overlapping work between primary and secondary care [2]. Delays caused by the diverse interfaces of care levels potentially result in worsened treatment outcomes and dissatisfaction among both patients and professionals [3–5]. Continuity of care is often heavily disturbed or non-existent when a patient moves between different care levels [6]. To control healthcare costs, gatekeeping (i.e. a general practitioner [GP] acting as a gatekeeper to specialised care) is being applied, but this strategy is not trouble-free either. It has been proven to control expenditure, but evidence of outcomes remains controversial [7–9].

Some results have shown that models of vertically integrated care may enhance patient satisfaction and perceived quality and improve access [10, 11]. A meta-analysis published in 2020 [12] showed a significant decrease in costs and an improvement in outcomes when integrated care is utilised. However, there is also evidence to the contrary, especially in terms of costs [13–15].

Numerous research articles have been published on healthcare integration, and there have been some reviews of an outreach model in which a hospital specialist visits primary care [13, 15–18]. In previous reviews, only a few studies have measured cost-effectiveness or reported on clinical outcomes, patient-reported outcome measures (PROMs), patient satisfaction or professional satisfaction simultaneously with costs. In terms of the studies that have been conducted, the results are conflicting, and the level of cost-effectiveness remains unclear [12, 19]. Moreover, professional aspects have seldom been included in previous reviews.

In this review, we concentrate on vertical integration in a model in which a hospital specialist visits primary care to determine whether it is cost-effective for a hospital specialist to see patients in a primary care setting instead of a hospital clinic. The question is surveyed via the concept of the Quadruple Aim [20]. The Quadruple Aim was expanded in 2014 from the concept of the Triple Aim—a framework to optimise healthcare system performance, encompassing cost reduction, population health improvement and patient experience improvement—by adding a fourth domain: healthcare professional well-being or satisfaction [20, 21]. The domains of population health, patient experience and healthcare professional well-being or satisfaction are considered to indicate quality in this review.

The primary objective of this systematic review is to determine whether specialist care can be brought to a primary care setting cost-effectively, with cost-effectiveness being the primary outcome. A secondary objective is to assess cost-effectiveness by comparing simultaneous changes in quality (health outcomes, patient experience, professional satisfaction) and cost. As a tertiary objective, changes in the aforementioned cost and quality parameters are analysed one by one.

## Methods

We conducted a systematic review and reported it following the Preferred Reporting Items for Systematic Reviews and Meta-Analyses (PRISMA) 2020 guidelines [22]. The protocol for this systematic review was registered on PROSPERO (CRD42022325232).

### Inclusion and exclusion criteria

A study was eligible for this review if it met the following criteria: (1) it involved a hospital specialist physician working in a general practice setting (also called an ‘outreach clinic’), (2) outcomes reported included both quality and costs on some level (at least one parameter of quality and either total cost or at least one cost driver) and (3) the specialty was somatic. In the context of this paper, *quality* refers to health outcomes, patient experience and professional satisfaction—that is, any non-cost-related outcomes of the intervention affecting either the patient or the professional. The specialist had to visit the primary care setting physically; e-consultations were excluded. We also excluded purely qualitative studies but included mixed-methods studies if they met the criteria delineated above. We also excluded psychiatry, substance abuse and dental/oral health care, which are often practised in separate units from the main primary and secondary care; thus, they deserve their own reviews concentrating on the relevant issues of the speciality. Furthermore, we only included studies published in full text in peer-reviewed journals. Some papers focused on either quality or cost, reporting the other only superficially (e.g. ‘cost was unchanged’). We chose to include these studies if they still reported the direction of change for both cost and quality.

### Literature search strategy

We searched for papers in the PubMed, Scopus, and EBSCO (CINAHL and Academic Search Complete) electronic databases. We included original studies published in English between 1.1.1992 and 4.2.2022. The selection of the electronic databases and a structured search strategy were developed with the help of an information specialist, and this included terms relating to specialised healthcare services at the primary healthcare level. The search strategy, search terms and variations for each database can be found in Additional file 1.

### Screening

All eligible studies were imported into the Covidence [23] tool for screening. Two reviewers (ML and LP) screened each study independently, first by title and abstract, and finally, by full text. After each step, the reviewers discussed possible disagreements and reconciled them by consensus; possible conflicts were resolved by the other researchers. Endnote 20.4.1 [24] was used as a reference manager.

### Assessment of quality and risk of bias

Quality assessment scores were calculated for all studies that otherwise fulfilled the inclusion criteria described above. We categorised the studies according to their methodology and assessed their quality using the Joanna Briggs Institute (JBI) quality appraisal checklists [25]. The studies were assessed for quality by one reviewer and double-checked for consistency by another. No automated tools were used in this process. Since there is no consensus on what constitutes sufficient quality when using the JBI checklist, a 50% minimum score was required for acceptance in this review. The completed checklists are available as Additional file 2.

We assessed the risk of bias for each study using the Risk Of Bias In Non-randomised Studies - of Interventions (ROBINS-I) tool [26]. Two reviewers independently completed the assessment, and consensus was then reached by discussion. The ROBINS-I results were visualised as a traffic light graph via Robvis software [27]. Studies with a critical risk of bias were left out of this review, as instructed in the ROBINS-I guidelines [26]. Studies included in this review after the quality check, were treated with similar importance, but detailed discussion was carried out where needed considering the risk of bias.

### Data extraction and synthesis

The cost-effectiveness of the intervention represents our primary interest, objective and outcome. Cost-effectiveness can be reported as the incremental cost-effectiveness ratio (ICER) [28]. The secondary outcomes are divided into the patient, professional and commissioner perspectives; they are presented using the Quadruple Aim, which includes the following: (1) population health, which covers health outcomes, both clinical (e.g. lowered blood pressure or biomarkers like blood cholesterol) and patient reported (including standardised quality-of-life questionnaires, both general and disease-specific); (2) patient experience, which covers patient-reported experience measures (PREMs) and such process parameters as wait times; (3) professional satisfaction; and (4) cost of care. Because outcome measures varied considerably between studies, study results other than cost-effectiveness were further grouped under the nine broader outcomes (O1–9), which are presented in Table 1.

**Table 1:** Secondary outcomes from the Quadruple Aim

**Quadruple Aim: main categories of outcomes**	**Outcome number**	**Secondary outcomes in this review**	**Examples of outcomes**
**1) Population health**	O1	Clinical outcomes	Biomarkers, such as blood cholesterol, or blood pressure (BP)
	O2	PROM	Standardised quality-of-life questionnaires, generic and disease specific
**2) Patient experience**	O3	PREM	Patient-reported experience measures, such as Net promoter score (NPS), as reported by patients
	O4	Process outcomes	Wait times
**3) Professional satisfaction**	O5	Professional satisfaction	Professional satisfaction questionnaires, interviews and other measures
**4) Costs**	O6	Monetary costs commissioner	Costs reported in some currency
	O7	Cost drivers commissioner	Non-attendance rates, referrals to the hospital/specialist or a follow-up specialist visit, laboratory referrals, imaging or other diagnostic examinations, expenditure of health services and so on
	O8	Monetary costs patient	Costs reported in some currency
	O9	Cost drivers patient	Travel time or distance, absence from work and so on

Categories 1 to 3 are referred to as ‘quality’ in this review. Cost parameters are categorised into monetary costs (reported in currency) and cost drivers, which are reported separately for commissioners and patients. Cost drivers refer to any parameters that directly affect monetary costs. Cost drivers for commissioners include non-attendance rates, referrals to a hospital/specialist or a follow-up specialist visit, laboratory referrals, imaging or other diagnostic examinations, expenditure on health services and so on, whereas cost drivers for patients include travel time or distance, absence from work and so on.

For the data extraction, templates were used to ensure that the approach was consistent with the research questions. The data was extracted by one reviewer (ML) and double-checked for consistency by another (LP). Extracted data included study details (author, year, country, intervention type, control group, medical specialties involved, population/sample size), methodology used, primary outcome (cost-effectiveness) and secondary outcomes for quality and costs, as described above.

For the synthesis of the data, meta-analysis was preferred. If this was not possible, the results would be narratively synthetised. The synthesis reporting was conducted by applying the Synthesis Without Meta-analysis (SWiM) [29] and Cochrane [30] guidelines to guarantee the quality of the narrative synthesis [31].

We explored heterogeneity using tables in comparing study designs, populations, interventions, outcomes and measures. In the case of the heterogeneity of the studies, following Boon and Thomson, vote counting based on direction of effect was used to investigate whether the intervention had any effect on the outcomes selected [30, 32]. In practice, where multiple measured parameters for one outcome within a study all report effects in the same direction, the effect direction is reported for the outcome (domain). Where the direction of effect varies across multiple measured parameters for an outcome within a study, the direction of effect where a minimum 70% (i.e. a clear majority) of parameters report similar directions is reported. If <70% of parameters report a consistent direction of effect, then no clear effect/conflicting/inconsistent findings are reported. An upward arrow (▲) indicates a positive (wanted) impact, a downward arrow (▼) represents a negative impact and a sideways arrow (◄►) indicates no change/mixed effects/conflicting or inconsistent findings. An empty cell in the table signifies that no outcomes in that category were measured in the study. The study population size is depicted in the size of the arrow: a large arrow  indicates a size of > 300, a medium arrow  indicates a size of 50–300 and a small arrow  indicates a size of < 50. The superscript by the arrow, if any, indicates the number of outcomes summarised in the direction of the arrow.

An effect direction plot, representing a tabulated summary of the direction of all reported impacts, was to be used to visualise the result of vote counting across the outcome domains [32]. The risk of bias was marked in its own column.

The sign test was utilised to provide statistical support for the synthesis of effect direction across studies for outcomes and to judge whether there was evidence of an effect. The sign test is a non-parametric test that uses a binary measure of either a positive or negative effect to test whether there is adequate evidence to reject the null hypothesis of an equal amount of positive and negative findings [33]. Studies with an inconsistent effect direction for an outcome are excluded from the sign test; this is because they do not represent either of the binary directions. The p-value of the sign test shows the probability of observing the given number of positive and negative findings if the null hypothesis were true [32].

For the secondary objective, considering the simultaneous change in cost and quality, a vote count for the ‘total quality’ and ‘total cost’ outcomes was summarised based on the original data and effect direction plot columns formulated according to the previously depicted principles. To avoid bias of double summation of the results in vote counting, the effect directions were summarised separately for each secondary outcome (O1–O9), for ‘total quality’ and ‘total cost’, as well as for the ‘total costs (commissioner)’ from the original data.

Three cross-tabulations of 3 × 3 were composed to present three different economic aspects over the total quality as follows: (1) all reported costs for both commissioners and patients (Table 4), (2) commissioners’ monetary cost and cost drivers (Table 5) and (3) commissioners’ monetary costs (Table 6). Cost is marked in the vertical and quality in the horizontal direction; both may be categorised as positive, negative, or inconsistent/no effect. A bar chart was drawn to visualise the results of these cross-tabulations.

 Finally, the strength of evidence for the reported primary outcome and Quadruple Aim outcome domains across the included studies were evaluated using the Grading of Recommendations Assessment, Development and Evaluation (GRADE) approach [34] with the GRADEPRO tool [35].

## Results

### Study selection

**Fig. 1:**
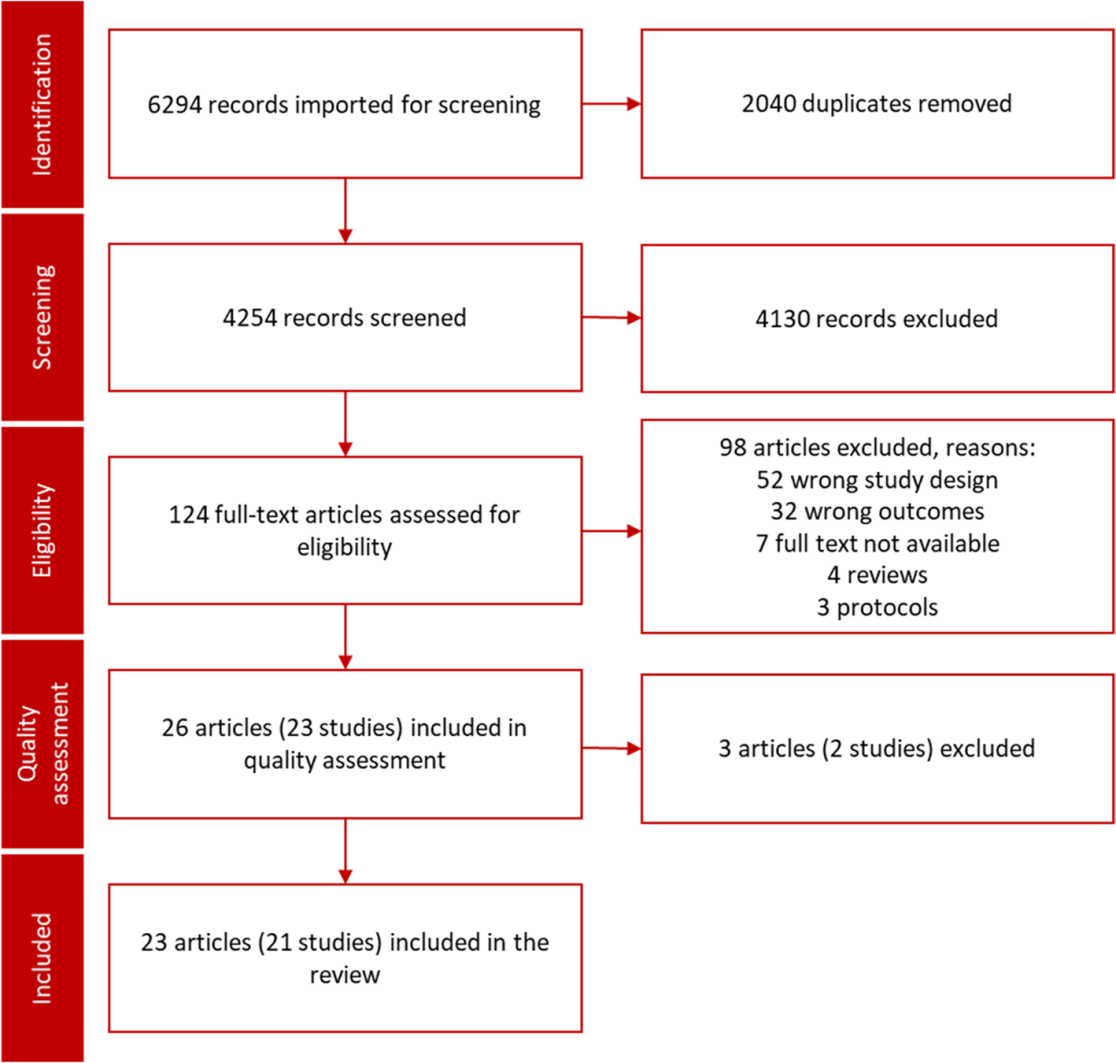
PRISMA flow chart of the study’s search strategy

The initial database search identified 6294 records. After duplicate removal, the number of records was 4254. Following elimination based on titles and abstracts, the full texts of 124 reports were selected for analysis. Seven full texts were not available; one of these [36] seemed to have the right setup for this review, the length of the article was three pages which may indicate challenges in the report quality required. The reports of Gosden et al. [37] and Black et al. [38] were from the same study, as were those of Dashora et al. from 2011 [39] and Dashora et al. from 2015 [40]. The report of Bowling et al. [41] included the population of Bond et al. [42]. Reports presenting the same study were combined as one row representing the study in the tables of data extraction and synthesis. In total, 26 reports representing 23 studies were included in the quality check [26]. The PRISMA flow chart of the study’s search strategy is shown in the Fig. 1.

**Fig. 2:**
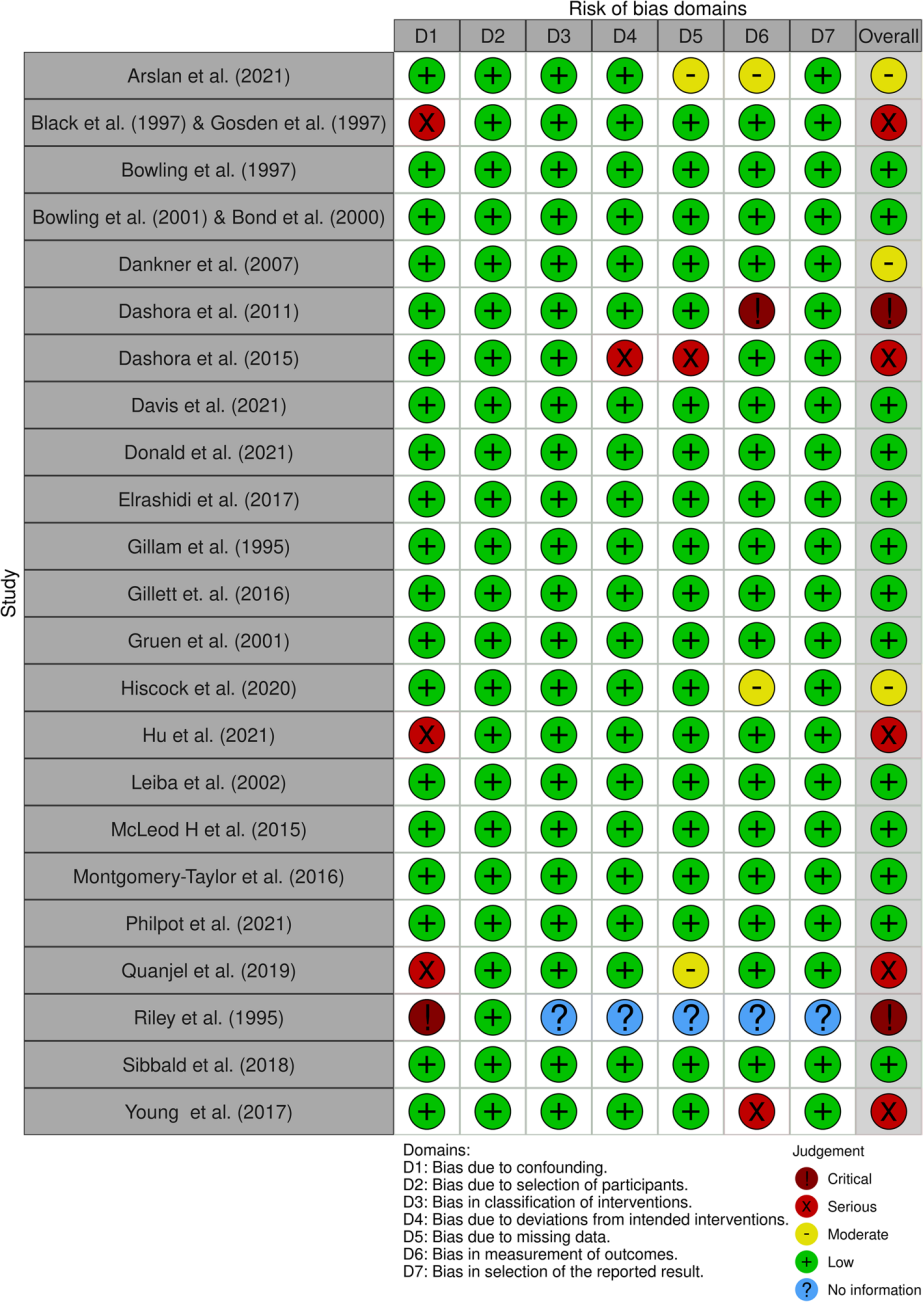
Summary of the ROBINS-I risk of bias classification by study

Three reports—those of Riley et al. from 1996 [43], Dashora et al. from 2011 [39] and Dashora et al. from 2015 [40]—were deemed to have a critical risk of bias by the ROBINS-I tool (Fig. 2). The same studies received scores of under 50% in the JBI appraisal (see Supplementary File 3 for full scores), and they were left out of this study. ﻿The report by Riley et al. from 1996 [43] had the right setup, but the description was scanty, as it was missing descriptions of the participants, follow-up and statistical testing. Dashora et al.’s 2015 study [40] lacked a control group, the follow-up was not complete and there was information missing, leaving uncertainty about many issues. Thus, these three sources were excluded from this review. Hence, 23 reports from 21 studies were included in this review. Considering all 21 studies that were evaluated, the most common sources of bias were missing data, confounding factors and measurement of outcomes (Fig. 2).﻿

### Study characteristics

Sample sizes of the studies ranged from 55 [44] to over 200 000 patients [45]. Four [46–49] of the 21 studies reported only the number of visits, number of professionals or both, but they did not report the number of patients attending. The most common medical specialities involved were orthopaedics (*n =* 7), gynaecology (*n =* 8), and general surgery (*n =* 6). In most studies, the intervention population, that is, patients visiting a specialist outside the hospital, was compared with the population attending a hospital outpatient clinic (*n =* 10), or with the same population pre-intervention (*n =* 7). Comparisons were made with both the pre-intervention population and a hospital outpatient group in two studies, with the units without outreach specialists in one study, specialised referral practice in one study and regular care in general practice in one study. In addition, professionals’ views were studied through interviews or questionnaires in 10 studies.

Studies were sorted according to the research design in Table 2, as quality appraisals differed in the different designs. Of the 21 studies, 3 received a full score on the JBI checklist, whereas 13 were graded as having a low risk of bias by the ROBINS-I tool. The denominator of the JBI assessment score represents the number of relevant questions in the checklist.

**Table 2:** Characteristics of the studies

**Reference number**	**Author (year)**	**Country**	**Intervention**	**Control group**	**Medical specialties involved**	**Population /Sample size**	**Method**	**JBI Quality assessment**	**ROBINS-I risk of bias assessment**
**Non-RCT or quasi-experimental studies, JBI Checklist *n =* 10**									
[46]	Dankner et al. (2007)	Israel	Hospital specialist outreach clinic in primary care (soldiers)	Soldiers’ primary care without specialists	Internal medicine, surgery, orthopaedics, dermatology, emergency medicine	4970 visits	Mixed	7/9	Moderate
[50]	Davis et al. (2021)	Australia	Hospital specialist consulting in general practice; upskilled GPs co-located	Pre-intervention patients	Endocrinology (diabetology)	213 patients	Mixed	8/9	Low
[51]	Gillam et al. (1995)	United Kingdom	Hospital specialist outreach clinic in general practice	Other GP practices and hospital outpatient clinic	Ophthalmology	1309 patients 55 GPs	Mixed	6/9	Low
[44]	Gillett et. al. (2016)	United Kingdom	Joint clinic in GP practice with hospital specialist	Pre-intervention patients	Pulmonology	55 patients	Mixed	8/9	Low
[52]	Hiscock et al. (2020)	Australia	Joint clinic in GP practice with hospital specialist	Pre-intervention patients	Paediatrics	284 patients 24 GPs	Mixed	6/9	Moderate
[53]	Montgomery-Taylor et al. (2016)	United Kingdom	Joint clinic in general practice with hospital specialist, GPs and multidisciplinary teams	Other GP practices and hospital outpatient clinic	Paediatrics	126 patients	Mixed	5/9	Low
[54]	Quanjel et al. (2019)	Netherlands	Hospital specialist in primary care (so-called Primary Care+)	Hospital outpatients	Cardiology	370 intervention patients, 291 controls	Mixed	8/9	Serious
[43]	Riley et al. (1996)	United Kingdom	Hospital specialist in primary care	Hospital outpatients	Gynaecology, orthopaedics and urology	Nearly 200 patient attendances	Mixed	4/9	Critical
[39, 40]	Dashora et al. (2011) Dashora et al. (2015)	United Kingdom	Joint clinic in general practice with hospital specialist, GP and multidisciplinary team	Pre-intervention patients	Endocrinology (diabetology)	15	Mixed	5/9 4/9	Critical
[55]	Young et al. (2017)	USA, Minnesota	Hospital specialist in primary care	Pre-intervention patients	Neurology	359 patients	Quantitative	5/9	Serious
**Cohort studies, JBI checklist, *n =* 6**									
[37, 38]	Black et al. (1997) Gosden et al. (1997)^a^	United Kingdom	Hospital specialist outreach clinic in general practice	Hospital outpatients	Dermatology, orthopaedics	164/242 patients 6 GPs, 6 specialists and managers in GP and hospital finance department	Mixed	6/8 6/8	Serious Serious
[56]	Bowling et al. (1997)	United Kingdom	Hospital specialist outreach clinic in general practice	Hospital outpatients	ENT, surgery, rheumatology and gynaecology	294 patients 9 specialists 44 GPs 9 practice managers	Mixed	6/8	Low
[41, 42]	Bowling et al. (2001) and Bond et al. 2000^b^	United Kingdom	Hospital specialist outreach clinic in general practice	Hospital outpatients	Cardiology, ENT, general medicine, general surgery, gynaecology, paediatrics and rheumatology	2344 patients 37 specialists 114 GPs 31 outpatient practice managers 26 outpatient trust accountants	Mixed	9/10 6/8	Low Low
[45]	Hu et al. (2021)	China	Precise care by a hospital specialist, GP and manager for DM and BP patients	Patients with regular care or no care	Internal medicine	154 651 patients with hypertension and 50 722 patients with diabetes	Quantitative	8/9	Serious
[49]	Leiba et al. (2002)	Israel	Hospital specialist outreach clinic in primary care for soldiers	Pre-intervention patients and a similar clinic employing only GPs referring soldiers to military specialist centres or hospital outpatient clinics	Internal medicine, general surgery, ENT, gynaecology, orthopaedics, neurology	11 GPs 7012 visits (Population not reported)	Mixed	8/10	Low
[57]	McLeod et al. (2015)	United Kingdom	Hospital specialist satellite clinics in community settings	Hospital outpatients	Paediatrics	Not reported	Quantitative	7/9	Low
**Case-control studies, JBI checklist, *n =* 2**									
[58]	Elrashidi et al. (2017)	USA, Minnesota	Hospital specialist outreach clinic in general practice	Non- co-located neurology patients	Neurology	459 cases 459 controls	Quantitative	9/10	Low
[59]	Philpot et al. (2021)	USA, Minnesota	Hospital specialist in the community	Pre-intervention patients	Gastroenterology	265 intervention patients 530 historical controls	Quantitative	10/10	Low
**Analytical cross-sectional studies, JBI checklist, *n =* 3**									
[48]	Gruen et al. (2001)	Australia	Hospital specialist outreach clinic in general practice	Hospital outpatients and pre-intervention patients	ENT, general surgery, gynaecology and ophthalmology	1979 gynaecologists 1430 ophthalmologists 1270 surgeons 275 ENT consultations	Mixed	5/8	Low
[60]	Gruen et al. (2006)	Australia	Hospital specialist outreach clinic in general practice	Hospital outpatients and no regular outreach	ENT, general surgery, gynaecology and ophthalmology	2368 patients	Quantitative	8/8	Low
[61]	Sibbald et al. (2008)	United Kingdom	Hospital specialist in the community ‘Closer to Home’ settings	Hospital outpatients	Dermatology, orthopaedics, gynaecology	783 intervention patients 275 control patients 58 commissioners, GPs, care providers and hospital specialists	Mixed	6/8	Low
**Case series, JBI checklist, *n =* 1**									
[62]	Arslan et al. (2021)	Netherlands	Hospital specialist working in general practice	Pre-intervention patients	Orthopaedics	96 control patients 208 intervention patients 7 professionals	Mixed	7/10	Moderate
**Economic evaluation, JBI checklist, *n =* 1**									
[47]	Donald et al. (2021)	Australia	Hospital specialist consulting GP with special interest in primary care	Hospital outpatients	Endocrinology (diabetology)	Clinic managers in GP practices and control hospitals; no number of patients reported	Mixed	11/11	Low
**Qualitative studies, JBI checklist, *n =* 5. All were mixed-methods studies and were evaluated according to another checklist above.**									
[62]	Arslan et al. (2021)	Netherlands	Hospital specialist working in general practice	Pre-intervention patients	Orthopaedics	96 control patients 208 intervention patients 7 professionals	Mixed	6/10	Moderate
[48]	Gruen et al. (2001)	Australia	Hospital specialist outreach clinic in general practice	Hospital outpatients and pre-intervention patients	ENT, general surgery, gynaecology and ophthalmology	1979 gynaecologists 1430 ophthalmologists 1270 surgeons 275 ENT visits 2368 surgical patients	Mixed	7/10	Low
[60]	Gruen et al. (2006)	Australia	Hospital specialist outreach clinic in general practice	Hospital outpatients and pre-intervention patients	ENT, general surgery, gynaecology and ophthalmology	2368 surgical patients	Mixed	7/10	Low
[53]	Montgomery-Taylor et al. (2016)	United Kingdom	Joint clinic in general practice with hospital specialist, GPs and multidisciplinary teams	Other GP practices and hospital outpatient clinic	Paediatrics	126 patients	Mixed	6/10	Low
[61]	Sibbald et al. (2008)	United Kingdom	Hospital specialist in the community ‘Closer to Home’ settings	Hospital outpatients	Dermatology, orthopaedics, gynaecology	783 intervention patients 275 control patients 58 commissioners, GPs, care providers and hospital specialists	Mixed	8/10	Low

*ENT* Ear-nose-throat, *GP* general practitioner, *DM* diabetes mellitus, *BP* blood pressure, *PREM* patient-reported experience measure

^a^Gosden et al. [37] and Black et al. [38] reported on the same study; the population in the wider economic analysis produced by Gosden et al. included 242 patients, whereas 164 patients completed the quality questionnaires on which Black et al. reported. Answers from six consultants were included in Gosden et al.’s report

^b^Bowling et al. [41] and Bond et al. [42] reported on the same study

### Primary objective: cost-effectiveness

Cost-effectiveness was calculated in 2 of the 21 studies. In an intervention involving lung diseases, in 2016, Gillet et al. [44] showed that the ICER (intervention vs no intervention) was 142.89 pounds sterling (£) per exacerbation of chronic obstructive pulmonary disease (COPD) that was avoided. In the study, cost drivers for the commissioner were lower, clinical outcomes improved, professionals rated the intervention extremely positively and all patients were satisfied with the intervention. In 2021, Donald et al. [47] found that, in the integrated care model for complex type II diabetes patients, the incremental cost savings were 365 Australian dollars (A$) per patient course of treatment compared with usual care for equivalent clinical outcomes. In the intervention, there was a higher number of visits featuring improved patient access, real-time follow-up and higher patient satisfaction. The risk of bias for both studies was low. According to the GRADE approach, the certainty of evidence for the finding is low, downgraded by one level for non–randomised controlled trial (RCT) study design and another level for imprecision because of the small population and inexact reporting of population size behind the outcome.

### Secondary objective: simultaneous changes in quality and cost as a proxy for cost-effectiveness

Table 3 presents the studies sorted in descending order based on the positiveness of the results. Studies of the same order are listed alphabetically. The effect direction plot with arrows illustrates the effect direction by the intervention (based on the vote count) on summarized outcomes 'Total quality, ‘Total cost’ and ‘Total cost commissioner’, as well as on primary and secondary outcomes. Superscripts beside the arrows indicate the number of secondary outcome categories summarized. Table 4 shows the studies cross-tabulated according to the effect directions on summarised quality and total cost. We will first discuss the category in which the effect direction on both quality and cost is positive. Next, we will examine studies where either cost or quality has a positive effect while the other parameter conflicts. Lastly, we will analyse studies in which both parameters conflict.

**Table 3:**
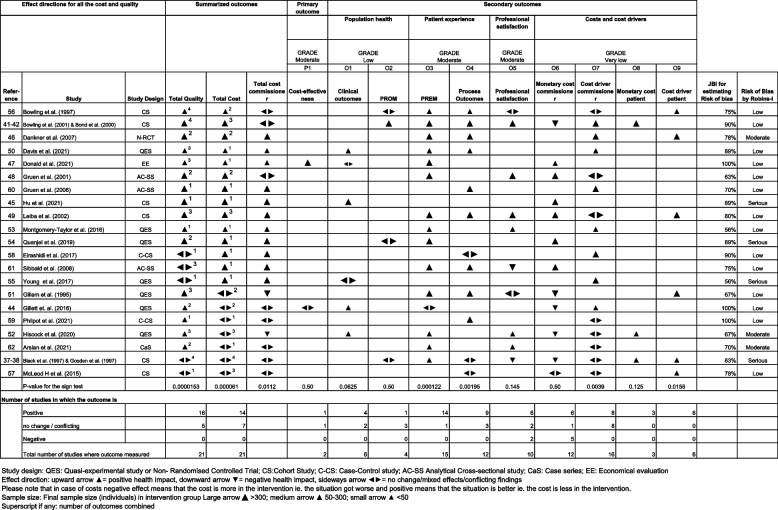
Effect direction plot of outcomes under the Quadruple Aim by study

**Table 4:** Studies according to the effect directions of summarised quality and total cost

		**Quality**		
		Positive	No change /conflicting findings	Negative
**Costs and cost drivers for patients and commissioners**	Positive	*n =* 11^a^	*n =* 3^c^	*n =* 0
	No change /conflicting findings	*n =* 5^b^	*n =* 2^d^	*n =* 0
	Negative	*n =* 0	*n =* 0	*n =* 0

^a^ [41, 45–50, 53, 54, 56, 60]

^b^ [44, 51, 52, 59, 62]

^c^ [55, 58, 61]

^d^ [37, 38, 57]

In 52% (*n =* 11) of the studies (Tables 3 and 4), the intervention direction of effect both on the quality (health outcomes, patient experience or professional satisfaction) and the costs, including cost drivers, was positive i.e. favouring the intervention. The single outcome parameters (O1-9) inside the summarised ones at least remained at the previous level, but mostly improved; the exception was the commissioner’s monetary cost in the study of Bowling et al. [41, 42], in which the effect was negative. In this study [41, 42], the intervention had a positive effect on the commissioner cost drivers (lower non-attendance rates, fewer follow-ups) and cost for the patient. Monetary costs for the patient were only measured in one [41, 42] of the 11 studies and cost drivers in three studies [46, 49, 56]. However, where measured, they were all lower in the intervention group; in three of four studies, they were significantly lower [41, 42, 49, 56]. Professional satisfaction was reported in 45% (*n =* 5) [41, 42, 48, 49, 53, 56] of these studies, 80% of which showed a positive change in satisfaction in the intervention group; one study [56] remained conflicting. In two of the previous studies, the risk of bias was serious.

In 38% (*n*=8) of the studies [44, 51, 52, 59, 62, 55, 58, 61], the effect on either quality or cost was positive; and on the other one the effect was conflicting or there was no change (see Tables 3 and 4). In five of these studies [44, 51, 52, 59, 62], the effect on quality measured via patient satisfaction, process outcomes and clinical outcomes, were positive (Table 3); for cost, the effects were conflicting. In the studies of Gillam et al. [51] and Gillet et al. [44], the intervention effect on the commissioner’s monetary cost was negative, but the measured cost drivers were positive. In the study by Hiscock et al. [52], the effect on the commissioner’s monetary cost was negative, whereas cost drivers were conflicting and the patient’s monetary cost positive. Two studies [59, 62] did not report monetary costs, and the effect direction to the cost drivers remained conflicting. Professional satisfaction, measured in three of eight studies, showed a positive effect direction in two studies [52, 62], and remained inconclusive in one [51]. In three studies [55, 58, 61], the effect on the quality (mainly wait times) remained conflicting, while for cost the effect direction was positive (only commissioner’s monetary cost or cost drivers reported).

In 10% (*n*=2), in the two least positive studies [37, 38, 57] of Tables 3 and 4, the effect directions of the intervention on both quality and cost were conflicting. The study by Black, which was described in two articles [37, 38], reported lower monetary costs for health services in dermatology; the results remained unclear for orthopaedics, but higher marginal monetary costs were found in both specialties in the intervention. By contrast, there were savings caused by significantly fewer patients called for follow-ups and fewer tests taken within dermatology. In addition, there were large but not significant differences in favour of outreach in terms of patient travel, time and total patient costs (reported in £). Simultaneously, the study reported better patient satisfaction but conflicting results for PROMs and process outcomes, as well as negative effects on professional satisfaction. The study reported many uncertainties in calculating costs. Moreover, there was a serious risk of bias in this study due to a potential confounding problem. In the study by McLeod et al. [57], the monetary costs for the commissioner of the two paediatric outreach clinics were opposite. Moreover, cost drivers did not significantly differ from the control clinics, but for the patient, cost drivers were positive because of the shorter travel distances.

In total, in 19 studies, the effect direction on the cost or quality was positive whereas the on the other one of these, no change, conflicting or positive effect direction was found in the vote counting (Table 3).

When only the commissioner cost drivers and monetary cost were considered against quality (Table 5), the effect direction was positive for both cost and quality in 38% (*n =* 8) of the studies; furthermore, either cost, or quality was positive and the other one showed conflicting findings in 43% (*n =* 9) of the studies. In 10% (*n =* 2) of the studies, the effect direction remained completely conflicting; and finally, in 10% (*n =* 2) of the studies, the quality was positive, but the cost was negative. The result is shown in the effect direction plot in Table 3 in the columns ‘Total cost commissioner’ and ‘Total quality’, while the number of studies in each category is cross-tabulated in Table 5.

**Table 5:** Studies according to the effect directions of summarised quality versus total cost for the commissioner

		**Quality**		
		Positive	No change /conflicting findings	Negative
**Total cost for commissioner**	Positive	*n =* 8^a^	*n =* 3^d^	*n =* 0
	No change /conflicting findings	*n =* 6^b^	*n =* 2^e^	*n =* 0
	Negative	*n =* 2^c^	*n =* 0	*n =* 0

^a^ [45–47, 49, 50, 53, 54, 60]

^b^ [41, 42, 44, 48, 56, 59, 62]

^c^ [51, 52]

^d^ [55, 58, 61]

^e^ [37, 38, 57]

In Table 5, ‘Total cost for commissioner’ indicates monetary cost and cost drivers (if both measured) for the commissioner, while ‘Quality’ indicates health outcomes, patient experience and professional satisfaction. The quality mostly improved (16/21), whereas the intervention effect on the costs or cost drivers was mostly positive (11/21) or conflicting (8/21) but seldom negative (2/12). In 17/21 studies either cost or quality improved while the other one at least remained or was inconsistent.

In Table 6, only monetary costs without cost drivers for the commissioner are considered with regard to quality (*n =* 12). In 50% (*n =* 6) of the studies in Table 6, the monetary cost for the commissioner was positive, as was the quality in five of these six studies. 42% (*n =* 5) of these studies (24% of the total of 21 studies) reported higher monetary costs for the commissioner. In two of these five studies [41, 42, 44], the cost drivers for the commissioner were positive, and in two studies [37, 38, 51], they were conflicting. The effects on the monetary costs or cost drivers for the patient were all positive, as was the quality in 75% of the studies; the rest remained conflicting.

**Table 6:** Studies according to the effect directions of summarised quality versus monetary cost for the commissioner

		**Quality**		
		Positive	No change /conflicting findings	Negative
**Monetary costs for commissioner**	Positive	*n =* 5^a^	*n =* 1^c^	*n =* 0
	No change /conflicting findings	*n =* 0	*n =* 1^d^	*n =* 0
	Negative	*n =* 4^b^	*n =* 1^e^	*n =* 0

^a^ [45, 47–49, 54]

^b^ [41, 42, 44, 51, 52]

^c^ [61]

^d^ [57]

^e^ [37, 38]

To visualise the comparison of quality against the previous aspects of cost in Tables 4, 5 and 6, a bar chart was drawn, see Supplementary Figure 1, Additional file 3.

The detailed results of the patient-reported measures and clinical measures are shown in Supplementary Table S1, Additional file 4. Costs and cost drivers are presented in detail in Supplementary Table S2, Additional file 4.

### Tertiary objective: the intervention effect on the secondary outcomes one by one

***The effect direction of the intervention considering monetary cost for the commissioner*** was positive in 29% (*n=*6) [45, 47–49, 54, 61], and negative in 24% (*n=*5) [37, 38, 41, 42, 44, 51, 52] of the studies. There was no clear evidence for either direction (*p =* 0.5). In 38% (*n=*8) of the studies [46, 50, 53, 55, 56, 58–60, 62], monetary costs were not reported at all. Risk of bias was serious in 2/6 of the studies with positive and in 1/5 of the studies with negative direction of effect (Table 3).

***The effect direction for the cost drivers for the commissioner*** (non-attendance rates, referrals and expenditure of health services) was positive in 38 % (*n=*8) [41, 42, 44, 46, 50, 53, 55, 58, 60] and inconclusive in 38% (*n=*8) of the studies [37, 38, 48, 49, 52, 56, 57, 59, 62], with no negative effect in any of the studies. The p-value for the sign test was 0.0039, favouring the intervention. ***For patients, the results for both monetary costs*** [37, 38, 41, 42, 52] ***and cost drivers*** [37, 38, 46, 49, 51, 56, 57] showed a positive direction; however, on the sign test, *p* < 0.05 was only reached for cost drivers. Reduced costs or cost drivers were also perceived as an advantage for the patient in all six interviews/questionnaires that covered the subject. The basis for the cost calculations was heterogeneous.

Because of the serious risk of bias, inconsistency, indirectness and imprecision detected in the GRADE appraisal, the rating of the certainty of evidence for the cost as a whole was downgraded to ‘very low’. A summary of the GRADE assessment of the certainty of evidence is presented in Supplementary Table S3, Additional file 5. The effect direction plot of the secondary outcomes sorted according to risk of bias can be found in Supplementary Table S4, Additional file 6.

***Clinical outcomes*** were reported in detail in 19% (*n=*4) [44, 45, 50, 52] of the studies, all improving by the intervention (*p =* 0.0625). Furthermore, 10% (*n=*2) [47, 55] of the studies reported no adverse outcomes and offered no further details. PROMs, including general and disease-specific standardised questionnaires, were utilised in four studies [38, 41, 42, 54, 56]. In one [41, 42] of these, the effect direction was positive, and the health status was significantly improved in the intervention group, whereas three studies [37, 38, 54, 56] showed inconsistent effect directions in the vote count. Together, clinical outcomes and standardised health questionnaires form a picture of population health (one aim of the Quadruple Aim framework). The certainty of the evidence for the intervention effect on health outcomes was assessed as ‘low’ with the GRADE approach. The rating was lowered because of the serious risk of bias and indirectness.

In 93% (*n=*14) of the 15 studies (67% of all the studies) that measured ***PREMs***, an intervention had a positive effect on patient satisfaction (*p =* 0.0001). In one study [44], the effect direction remained inconclusive. ***Process outcomes***—mainly wait times—were measured in 12 studies (57%). In nine (75%) of these [41, 42, 47, 49–52, 56, 59, 61], the effect direction was positive in favour of the intervention (*p =* 0.00195). In three (25%) [37, 38, 57, 58] the effect direction remained inconclusive. In addition to quantitative process outcome measures, wait times were reported in six interviews, with 83% of patients reporting shorter wait times for appointments in the intervention group. Professionals brought up the same advantage in three interviews.

Together, PREMs and process outcomes form a picture of **patient experience**, representing one aim of the Quadruple Aim. Except for imprecision, there was no need for downgrading the rating in the GRADE appraisal, resulting in a moderate certainty of evidence of the patient experience.

***Professional satisfaction*** was measured in 10 studies (48%). Six (60%) of these [41, 42, 48, 49, 52, 53, 62] showed a positive effect direction, whereas two (20%) [51, 56], were inconclusive and two (20%) [37, 38, 61, 37, 38, 61]were negative in the intervention group, one of which having a serious risk of bias [37, 38]. Professionals appreciated patients’ shorter wait times [38, 42, 56] and easier access to specialists [41, 42, 48, 56]. GPs appreciated the possibility of widening their skills, although some reported tight schedules hampering collaboration on site [38, 41, 48, 53, 56, 62]. In addition, specialists found the intervention to be a useful learning experience [51]. Specialists and managers appreciated the improved precision of referrals from GPs [42]. Both GPs and specialists appreciated improvement in communication and collaboration between GPs, remote clinics and specialists [42, 48, 53, 56, 62]. They noted that the setup promoted goodwill and developed social capital, trust and reciprocity between professionals [42, 53, 62]. One of the main disadvantages of outreach reported by GPs was infrequent or inflexible follow-up intervals, reflecting the frequency with which the clinic was held [41]. The main disadvantages reported by specialists focused on a reduction in specialists’ time in hospital [41, 42, 56, 61] and time spent travelling [38, 42, 56, 61]. Furthermore, some concerns were voiced regarding the diagnostic facilities in primary care [56, 62], and the patient mix became more difficult at the hospital clinics when the simpler cases were treated in primary care [61, 62]. Overall, professionals—both GPs and specialists—were more often satisfied, and they considered the model worthwhile [41, 42, 56].

In the GRADE appraisal, the rating was only downgraded because of serious indirectness. This resulted in a ‘moderate’ certainty of evidence for positive effect on professional satisfaction.

**Fig. 3:**
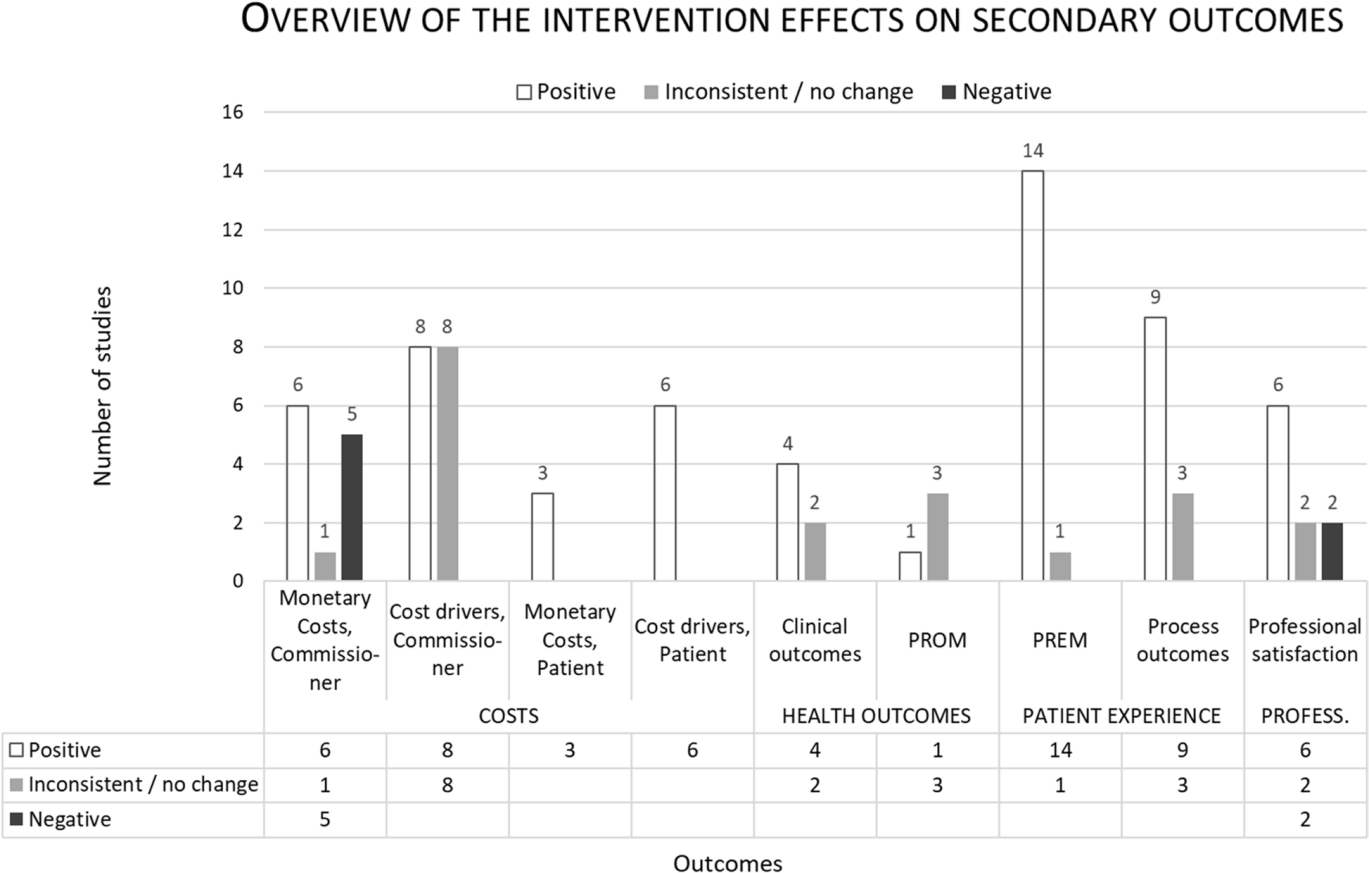
Overview of the intervention effects on secondary outcomes

Figure 3 presents an overall view of the results by measured variables—that is, defined secondary outcomes. The first column above each variable expresses the number of studies in which the results improved, while the second column shows the number of studies with the inconsistent results, and the third column shows the number of studies in which the result was negative in the intervention group.

## Discussion

The primary objective—cost-effectiveness of the specialist in primary care model—remains debatable, as it was reported on in only two studies [44, 47]. In one of these, the results were in favour of the intervention [47]; in the other [44], the conclusion depended on the acceptable threshold cost for the intervention—de facto, an acceptable threshold cost for COPD exacerbation—which is not known. None of the studies reported a loss of quality-adjusted life years (QALYs) or an increase of disability-adjusted life years (DALYs). There was also very limited information on cost-effectiveness in previous reviews [18, 19]. In the review by Sibbald [16], it was reported that the effectiveness and efficiency of the intervention depend on the location and previous service level in the primary care: in well-served urban populations, relocation of a specialist has been reported to be likely to diminish service effectiveness and efficiency, whereas services to underserved populations have tended to be more cost-effective.

As a proxy for cost-effectiveness the secondary objective of this review was to analyse the simultaneous change in cost and quality. Our study showed that, whereas monetary costs for the commissioner varied, the intervention had a positive effect on costs and cost drivers for the patient and a positive or inconclusive effect on cost drivers for the commissioner. Simultaneously, the parameters of health outcomes and patient experience at least remained the same or were inconclusive in any direction, but most often, they improved. Except for two studies, also the professional satisfaction improved or was inconclusive.

The conclusion of cost-effectiveness is still ambiguous. Uncertainty remains in terms of de facto effectiveness, because there are no commensurate parameters in the different categories of outcomes measured (costs, cost drivers, health and satisfaction) to be summarised. Moreover, because of the heterogeneity of the measured parameters and scales, we were unable to calculate a comparable ratio for the reported cost and quality parameters. The greatest uncertainty in the results may appear in studies where the opposite direction of effects among the summarised outcome parameters exists. In this review, this mostly appeared in the studies where the commissioner monetary cost effect direction was different from the drivers and patient cost, precluding a conclusion on the true effect direction on costs [37, 38, 41, 42, 51, 52]. For example, in the study of Bowling and Bond [41, 42], the negative effect on the commissioner’s monetary cost can be partly compensated, completely compensated or overcompensated for by the intervention’s positive effect on the cost drivers (lower non-attendance rates, fewer follow-ups) and lower cost for the patient. A lack of absolute outcome magnitudes and comparable units leaves the result (cost-effectiveness) of an intervention uncertain. Because the magnitudes of the effects are not known in the studies of Hiscock et al. and Gillam et al. either, the true economic effect remains unknown. Some studies clearly stated that costs reported by the organisations were not comparable; for example, Black [38] reported outright that ‘like was not comparable with like’. Furthermore, there were uncontrolled differences in the case mix.

Poor economic results of vertical integration interventions have been reported in many previous reviews and original articles [13–15]. Bringing specialists to primary care has been reported as more expensive [14, 15] or less efficient [18, 19] than specialised care in hospital outpatient clinics. However, in their 2003 Cochrane review, Gruen et al. [13] proposed that additional costs of outreach may be balanced by improved health outcomes. Although many of the previous reviews reported negative results in terms of the monetary costs for the commissioner, cost drivers or costs for the patient improved [15, 18], as also found in this review. The economic impact of cost drivers may be less straightforward, and their value has been partly left out in economic conclusions, which could be one reason for the previously reported economically unfavourable results for the outreach models. Including cost drivers may have led to a more positive result in this review.

Delving in detail into the causes of costs of the treatment models, a few problems can be observed. First, in some cases, the organisation of specialist outreach has not been optimal. For example, working days have been short, at half a day, or around 3 hours [42, 56, 57]. In specialist outreach clinics, the number of patients per specialist per day has been remarkably low, even down to one-third of that of outpatient clinics [37, 51]. Therefore, the travel time and expense of the specialist play a significant role in marginal costs [41, 42, 55]. These problems can usually be resolved through efficient management, which may involve various means such as carefully planning the specialist's schedule. Second, in some cases, the outreach clinics are staffed solely by consultants; unlike hospital clinics, where patients are seen either by consultants or registrars [37, 38], consultants charging higher salaries, resulting in higher costs in outreach clinics. Third, the cost and cost-effectiveness of the whole concept of specialists in primary care is apparently affected by the characteristics of the specialist. These include seniority [49], commitment, attitude, working efficiency and willingness to educate, and the unit price.

Further, the limitations and points of view of each study can substantially influence the results and conclusions drawn, including in this study. When only the commissioner’s costs were considered and cost drivers were ignored, the intervention setup seemed superior, but economically, the result did not seem as positive (Table 6).

Among the reviewed studies, we found that both monetary costs and cost drivers were lower for the patients in the intervention groups. The same result was found in all six interviews/questionnaires in which the topic was addressed, strengthening the quantitative result. Bringing specialists to primary care also seemed to be advantageous for the patient in other ways: patient-reported experience measures and quantitative process outcomes improved in over 93% of the studies measured and in 67% of all studies included in this review, which is consistent with previous reviews [13, 15, 16, 18]. Apart from the quantitative process outcome measures, reduced wait times were reported by the patient as an advantage in five of the six interviews. Professionals reported similar results in their interviews, thereby endorsing the quantitative results.

Health outcomes, although seldom measured, mainly improved [44, 45, 50, 52] in the studies, whereas in the previous reviews, they varied or were unclear [13, 16, 19, 63]. The results seem to vary by speciality and type of intervention [13]. In our study, although the clinical outcomes with change measured all showed a positive effect direction on vote counting, the p-value for the sign test remained over 0.05 (0.0625). The small number of studies (four) reporting the change of effect direction on outcome, may have partly affected this result, since the sign test requires a minimum of five measurements, all in the same direction, to reach statistical significance, if that is set to *p* < 0.05.

Professional satisfaction improved, where measured, in 60% of the studies. The GPs were generally very satisfied. Although some of the specialists had concerns, they mostly found the setup worthwhile. This result is noteworthy, as the opinions of professionals are crucial for the model’s continued success. Furthermore, given the threat of labour shortage and difficulties in recruiting personnel, especially in remote areas, the impact of a reasonable working environment and professional satisfaction must not be overlooked. Professionals’ satisfaction has rarely been reported on in previous reviews along with patient outcomes and economic analysis.

Sensitivity analysis based on risk of bias showed that leaving out studies with serious risk of bias by ROBINS-I [37, 38, 45, 54, 55], or the studies [37, 38, 52, 55, 62] that scored worse than average on both of the quality scores i.e. max. 75% in the JBI and either moderate, or serious risk of bias in the ROBINS-I, it would not have changed the direction of effect on outcomes, nor would it have changed the conclusions of this review.

### What do the studies in which all the measured Quadruple Aim simultaneously improved have in common?

In the setting of Leiba et al.’s study [49], the outreach clinic specialists were oriented towards primary medicine and unobligated to a large medical centre. Having seniority in their specialty, specialists took the role of ‘secondary gatekeepers’ and prevented unnecessary referrals to hospital specialists, as well as costly diagnostic tests and modalities. From these results, the proximity of consultants and specialists seems to be essential: it offers the advantage of non-formal medical interactions and on-the-job training, as well as an opportunity to improve clinical skills and professional capability, improve collaboration and professional relationships, and develop networks and social capital. As a result, the threshold for contact, questions, and clinical discussion is reduced, supporting the care of the patient in primary care by GPs [44, 49, 53].

Montgomery-Taylor et al. [53] suggested that the population for the outreach clinic needs to be large enough to be profitable; a population of 15 000–20 000 is proposed. In Leiba’s study [49], the patient volume was also remarkable, and the operation of the clinic was continuous. The same aspect was recognised in van Hoof et al.’s review [15]. Continuous or longer lasting operation is probably crucial for the setup to be able to optimise and develop its operation, considering all stakeholders. The high level of attendance suggests that patients may be more motivated to attend specialist clinics located in a primary care setting compared with their local hospital; thus, specialists in primary care could potentially provide a more acceptable, efficient and cost-effective service [44]. An outreach setup could potentially improve the equity of patients through better access [48, 51, 61, 62]. Indeed, equity has been considered to be added as a fifth domain to the Quadruple Aim, forming the Quintuple Aim [64].

### Strengths and limitations

As far as we know, this is the first review of implications of the outreach model that tracks both cost and quality implications simultaneously by study. The review spans 30 years, and over 4000 records were screened. Thus, the evidence base studied is wide. To increase the robustness of the review, we employed multiple systematic methods of quality appraisal.

No RCTs were found on the research topic, which possibly indicates the difficulty of running trials on system impact research [65]. The research setups were mainly observational cohort studies (*n =* 6) or quasi-experimental studies (*n =* 8), which could be seen as a negative factor for the review. However, for example, Quanjel et al. [54] showed that also practice-based observational research (non-RCT) can generate results that are generalisable and can easily be translated into practice. The pragmatic and economic issues of intervention study designs have been discussed previously, resulting in an approbative understanding of the non-RCT study settings in system impact research [65, 66].

The healthcare model interventions and measured outcome variables in the reviewed studies were heterogeneous, and estimating the commensurate value of satisfaction, process measures or health outcomes is complicated. This made it difficult to execute a statistical summary (meta-analysis) of the studies. Therefore, vote counting was chosen as a method of estimating the direction of the effects of the intervention. The weakness of this approach is that it does not provide information on the magnitude of effects, nor does it account for differences in the relative size or quality of the studies [67].

It was also partly unclear exactly what was included in the cost calculations of the studies. Considering the total cost, the certainty of evidence was very low according to the GRADE strength of evidence rating. Therefore, it is difficult to draw strong overall conclusions about the total costs.

In our study, there were also evaluations in which the quality [55, 58] was not detailed; instead, only an outcome was reported, making it impossible to evaluate that part of the study. Nevertheless, leaving these studies out of the research would not have changed the results of any part of the review.

Although the search protocol was wide, we could not be sure that all relevant studies were identified. The search protocol was formulated, run and double-checked with an informaticist, which probably decreased the risk of bias in the search procedure. In any case, the results should be considered alongside the literature that did not meet the criteria for this review; this would mainly include studies reporting either quality or costs, but not both. Furthermore, a publication bias of publishing more positive results cannot be excluded. Some of the included studies were reported to be pilots with limited populations and follow-up periods. None of the studies used a follow-up period of over 2 years; therefore, they missed the effects of both the positive impacts and potential challenges of long-term operation. Six studies dated back more than 20 years. Five reports of three studies were written by the same research group; in two of these, the monetary cost was reported to be higher in the intervention group.

### Implications for future research

To compare the true cost-effectiveness or total cost of the intervention and other healthcare operating models, the cost of the complete care path needs to be calculated, with clarity, stating the perspectives of different stakeholders.

We suggest using of a standardised cost breakdown. As one possibility, costs could be categorized as direct, indirect and intangible, as stated by Drummond et al. [68]), and ‘other’ costs, commonly omitted from cost-of-illness (COI) analyses, as proposed by Bugge et al. [69]. Further research is needed to establish such a standardised cost breakdown. Longitudinal comparative research, with a duration sufficient to comprehensively capture the perspectives of the different stakeholders—commissioners, patients and professionals, as intended by the Quadruple Aim—would provide clarity on the cost-effectiviness of the model.

## Conclusions

The cost-effectiveness of the hospital specialist in the primary care model remains unclear. However, it seems that there may be potential for organizing the model in a cost-effective manner for the commissioner. For the patient, the model appears to be superior. The cost is lower, access is better, patient satisfaction is high, and clinical and patient-reported outcome parameters mainly improve or remain the same. Accessibility and low costs can be seen as enhancing equity. Professionals, especially in primary care, appreciate the model, and despite some concerns, hospital specialists primarily view the model as worthwhile.

To calculate the cost-effectiveness of the service model, we propose a standardised cost division framework and longitudinal follow-up to be used in future interventions; and the value of equity in health care to be estimated. There is a severe risk of being misled if only the easily measured results (instead of the real cost and cost drivers) are considered in the calculation of the cost-effectiveness of the intervention.

### Supplementary Information

**Additional file 1. **Search Protocol for the systematic review.

**Additional file 2. **Joan Briggs Institute (JBI) quality appraisal of the studies.

**Additional file 3: Figure S1.** presents the simultaneous directions of effects on cost and quality following the specialist in primary care intervention. It visualises the comparison of quality against the different aspects of costs presented in Tables 4, 5 and 6 in the review. The first column above every category on horizontal axis represents the case in which all the costs and quality outcomes are taken into account (Table 4).The second column presents the case where monetary cost and cost drivers for the commissioner and all the quality outcomes are is taken into account (Table 5). The third column presents the case where only monetary cost for the commissioner against the quality is taken into account (Table 6). **Figure S1.** Simultaneous effect directions on cost and quality following the specialist in Primary care intervention.

**Additional file 4: Table S1.** includes Clinical outcomes and patient-reported outcome measures (PROMs) and **Table S2** includes detailed costs and cost drivers.

**Additional file 5: Table S3.** includes GRADE appraisal of the outcomes.

**Additional file 6: Table S4.** presents the ED plot of secondary outcomes sorted by Risk of Bias.

**Additional file 7. **The citation for the Synthesis Without Meta-analysis explanation and elaboration article is: Campbell M, McKenzie JE, Sowden A, Katikireddi SV, Brennan SE, Ellis S, Hartmann-Boyce J, Ryan R, Shepperd S, Thomas J, Welch V, Thomson H. Synthesis without meta-analysis (SWiM) in systematic reviews: reporting guideline BMJ 2020;368:l6890 http://dx.doi.org/10.1136/bmj.l6890.

## Data Availability

The datasets not included in the additional files but used and analysed during the current study are available from the corresponding author on reasonable request.

## Abbreviations

BP: Blood pressure
COI: Cost-of-illness
COPD: Chronic obstructive pulmonary disease
DALY: Disability-adjusted life year
DM: Diabetes mellitus
GP: General practitioner
ICER: Incremental cost-effectiveness ratio
JBI: Joanna Briggs Institute
PREM: Patient-reported experience measure
PRISMA: Preferred Reporting Items for Systematic Reviews and Meta-Analyses
PROM: Patient-reported outcome measure
QALY: Quality-adjusted life year
RCT: Randomised controlled trial
SWiM: Synthesis without meta-analysis

## References

[1] World health statistics 2022: monitoring health for the SDGs, sustainable development goals.: World Health Organization; 2022.

[2] Kern LM, Seirup JK, Casalino LP, Safford MM (2017). Healthcare Fragmentation and the Frequency of Radiology and Other Diagnostic Tests: A Cross-Sectional Study. J Gen Intern Med..

[3] Otto CM (2019). Heartbeat: Primary care delays in heart failure diagnosis. HEART..

[4] Pitkänen Sari YL (2020). Ihosyöpien diagnostiikka ja hoito: Kustannus Oy Duodecim.

[5] Vahti J. Sydänpotilaiden pirstaleinen polku paranisi asiakaslähtöisyydellä: SITRA; 2015 [cited 2022 6.8.2022]. Available from: https://www.sitra.fi/uutiset/sydanpotilaiden-pirstaleinen-polku-paranisi-asiakaslahtoisyydella/.

[6] Suomela Tuuli LI (2020). Rekisteritietoa seurantaan: 65 vuotta täyttäneiden hoidon jatkuvuus perusterveydenhuollossa. Lääkärilehti..

[7] Forrest CB (2003). Primary care in the United States: Primary care gatekeeping and referrals: effective filter or failed experiment?. BMJ..

[8] Garrido MV, Zentner A, Busse R (2011). The effects of gatekeeping: A systematic review of the literature. Scand J Prim Health Care..

[9] Sripa P, Hayhoe B, Garg P, Majeed A, Greenfield G (2019). Impact of GP gatekeeping on quality of care, and health outcomes, use, and expenditure: a systematic review. BR J GEN PRACT..

[10] Baxter S, Johnson M, Chambers D, Sutton A, Goyder E, Booth A (2018). Understanding new models of integrated care in developed countries: a systematic review. Health Serv Del Res.

[11] Strandberg-Larsen M (2011). Measuring integrated care. Dan Med Bull.

[12] Rocks S, Berntson D, Gil-Salmerón A, Kadu M, Ehrenberg N, Stein V (2020). Cost and effects of integrated care: a systematic literature review and meta-analysis. Eur J Health Econ.

[13] Gruen RL, Weeramanthri TS, Knight SE, Bailie RS (2004). Specialist outreach clinics in primary care and rural hospital settings. Cochrane Database Syst Rev..

[14] Powell J (2002). Systematic review of outreach clinics in primary care in the UK. J Health Serv Res Policy..

[15] van Hoof SJM, Quanjel TCC, Kroese M, Spreeuwenberg MD, Ruwaard D (2019). Substitution of outpatient hospital care with specialist care in the primary care setting: A systematic review on quality of care, health and costs. PLoS One..

[16] Sibbald B, McDonald R, Roland M (2007). Shifting care from hospitals to the community: a review of the evidence on quality and efficiency. J Health Serv Res Policy..

[17] Winpenny E, Miani C, Pitchforth E, Ball S, Nolte E, King S (2016). Outpatient services and primary care: scoping review, substudies and international comparisons. Health Serv Deliv Res.

[18] Winpenny EM, Miani C, Pitchforth E, King S, Roland M (2017). Improving the effectiveness and efficiency of outpatient services: a scoping review of interventions at the primary-secondary care interface. J Health Serv Res Policy..

[19] Machta RM, Maurer KA, Jones DJ, Furukawa MF, Rich EC (2019). A systematic review of vertical integration and quality of care, efficiency, and patient-centered outcomes. Health Care Manage Rev..

[20] Bodenheimer T, Sinsky C (2014). From triple to quadruple aim: care of the patient requires care of the provider. Ann Fam Med..

[21] Berwick DM, Nolan TW, Whittington J (2008). The triple aim: care, health, and cost. Health Aff (Millwood)..

[22] Page MJ, McKenzie JE, Bossuyt PM, Boutron I, Hoffmann TC, Mulrow CD (2021). The PRISMA 2020 statement: an updated guideline for reporting systematic reviews. BMJ..

[23] Covidence systematic review software. Veritas Health Innovation, Melbourne, Australia.

[24] Team Te (2013). Endnote. Endnote 20.4.1 ed.

[25] JBI Manual for Evidence Synthesis. Joanna Briggs Institute 2020. Available from: https://synthesismanual.jbi.global.

[26] Sterne JA, Hernán MA, Reeves BC, Savović J, Berkman ND, Viswanathan M (2016). ROBINS-I: a tool for assessing risk of bias in non-randomised studies of interventions. BMJ..

[27] McGuinness LA, Higgins JPT. Risk-of-bias VISualization (robvis): An R package and Shiny web app for visualizing risk-of-bias assessments. Research Synthesis Methods. 2020;n/a(n/a). 10.1002/jrsm.1411 32336025

[28] Incremental Cost-Effectiveness Ratio (ICER) [Internet]. York Health Economics Consortium. 2016. Available from: https://yhec.co.uk/glossary/incremental-cost-effectiveness-ratio-icer/.

[29] Campbell M, McKenzie JE, Sowden A, Katikireddi SV, Brennan SE, Ellis S (2020). Synthesis without meta-analysis (SWiM) in systematic reviews: reporting guideline. BMJ..

[30] Higgins JPT TJ, Chandler J, Cumpston M, Li T, Page MJ, Welch VA. Cochrane Handbook for Systematic Reviews of Interventions version 6.3. Cochrane. 2022. Available from: www.training.cochrane.org/handbook.

[31] Jennie P. Guidance on the Conduct of Narrative Synthesis in Systematic Reviews A Product from the ESRC Methods Programme. In: Helen R, editor. 2006.

[32] Boon MH, Thomson H (2020). The effect direction plot revisited: Application of the 2019 Cochrane Handbook guidance on alternative synthesis methods. Res Synth Methods..

[33] Moore DSMG (2002). Introduction to the Practice of Statistics.

[34] Schünemann H BJ, Guyatt G, Oxman A. GRADE handbook for grading quality of evidence and strength of recommendations: The GRADE Working Group, 2013; 2013.

[35] GRADEpro GDT: GRADEpro Guideline Development Tool [Software] (2022). McMaster University and Evidence Prime.

[36] Roland M, Sibbald B, McDonald R (2007). Care closer to home. Moving care. Health Serv J..

[37] Gosden T, Black M, Mead N, Leese B (1997). The efficiency of specialist outreach clinics in general practice: is further evaluation needed?. J Health Serv Res Policy..

[38] Black M, Leese B, Gosden T, Mead N (1997). Specialist outreach clinics in general practice: what do they offer?. Br J Gen Pract..

[39] Dashora U. Integrated care Improving glycaemic control in joint clinics. Diabetes Prim Care. 2011;13(6):369–74.

[40] Dashora U (2015). Ongoing benefit of improved control after a short-duration integrated joint clinic intervention in primary care. Diabetes Prim Care..

[41] Bowling A, Bond M (2001). A national evaluation of specialists' clinics in primary care settings. Br J Gen Pract..

[42] Bond M (2000). Evaluation of outreach clinics held by specialists in general practice in England. J Epidemiol Community Health..

[43] Riley K (1996). Outreach Clinics. Practice does not make perfect. Health Serv J..

[44] Gillett K, Lippiett K, Astles C, Longstaff J, Orlando R, Lin SX (2016). Managing complex respiratory patients in the community: an evaluation of a pilot integrated respiratory care service. BMJ Open Respir Res..

[45] Hu H, Liang H, Wang H (2021). Longitudinal study of the earliest pilot of tiered healthcare system reforms in China: Will the new type of chronic disease management be effective?. Soc Sci Med..

[46] Dankner R, Rieck J, Bentacur AG, Bar Dayan Y, Shahar A (2007). Civilian doctors in military clinics–outsourcing for better medicine. Mil Med..

[47] Donald M, Jackson CL, Byrnes J, Vaikuntam BP, Russell AW, Hollingworth SA (2021). Community-based integrated care versus hospital outpatient care for managing patients with complex type 2 diabetes: costing analysis. Aust Health Rev..

[48] Gruen RL, Bailie RS, d'Abbs PH, O'Rourke IC, O'Brien MM, Verma N (2001). Improving access to specialist care for remote Aboriginal communities: evaluation of a specialist outreach service. Med J Aust..

[49] Leiba A, Martonovits G, Magnezi R, Goldberg A, Carroll J, Benedek P (2002). Evaluation of a specialist outreach clinic in a primary healthcare setting: the effect of easy access to specialists. Clin Manage..

[50] Davis TME, Drinkwater JJ, Fegan PG, Chikkaveerappa K, Sillars B, Davis WA (2021). Community-based management of complex type 2 diabetes: adaptation of an integrated model of care in a general practice setting. Intern Med J..

[51] Gillam SJ, Ball M, Prasad M, Dunne H, Cohen S, Vafidis G (1995). Investigation of benefits and costs of an ophthalmic outreach clinic in general practice. Br J Gen Pract..

[52] Hiscock H (2020). Strenghtening care for children: pilot of an integrated general practitioner-paediatrician model of primary care in victoria, australia. Aust Health Rev.

[53] Montgomery-Taylor S, Watson M, Klaber R (2016). Child Health General Practice Hubs: a service evaluation. Arch Dis Child..

[54] Quanjel TCC, Spreeuwenberg MD, Struijs JN, Baan CA, Ruwaard D (2019). Substituting hospital-based outpatient cardiology care: The impact on quality, health and costs. PLoS One..

[55] Young NP, Elrashidi MY, Crane SJ, Ebbert JO (2017). Pilot of integrated, colocated neurology in a primary care medical home. J Eval Clin Pract..

[56] Bowling A, Stramer K, Dickinson E, Windsor J, Bond M (1997). Evaluation of specialists' outreach clinics in general practice in England: process and acceptability to patients, specialists, and general practitioners. J Epidemiol Community Health..

[57] McLeod H, Heath G, Cameron E, Debelle G, Cummins C (2015). Introducing consultant outpatient clinics to community settings to improve access to paediatrics: an observational impact study. BMJ Qual Saf..

[58] Elrashidi MY, Philpot LM, Young NP, Ramar P, Swanson KM, McKie PM (2017). Effect of integrated community neurology on utilization, diagnostic testing, and access. Neurol Clin Pract..

[59] Philpot LM, Ramar P, Sanchez W, Ebbert JO, Loftus CG (2021). Effect of Integrated Gastroenterology Specialists in a Primary Care Setting: a Retrospective Cohort Study. J Gen Intern Med..

[60] Gruen RL, Bailie RS, Wang Z, Heard S, O'Rourke IC (2006). Specialist outreach to isolated and disadvantaged communities: a population-based study. Lancet..

[61] Sibbald B, Pickard S, McLeod H, Reeves D, Mead N, Gemmell I (2008). Moving specialist care into the community: an initial evaluation. J Health Serv Res Policy..

[62] Arslan IG, Voorbrood VMI, Stitzinger SAG, van de Kerkhove MP, Rozendaal RM, van Middelkoop M (2021). Evaluation of intermediate care for knee and hip osteoarthritis: a mixed-methods study. BMC Fam Pract..

[63] Amado GC, Ferreira DC, Nunes AM (2022). Vertical integration in healthcare: What does literature say about improvements on quality, access, efficiency, and costs containment?. Int J Health Plann Manage..

[64] Dzau VJ, Mate K, O'Kane M (2022). Equity and Quality-Improving Health Care Delivery Requires Both. JAMA..

[65] Sanson-Fisher RW, Bonevski B, Green LW, D'Este C (2007). Limitations of the randomized controlled trial in evaluating population-based health interventions. Am J Prev Med..

[66] Kessler R, Glasgow RE (2011). A proposal to speed translation of healthcare research into practice: dramatic change is needed. Am J Prev Med..

[67] Borenstein MH, Larry; Higginns, Julian; Rothstein, Hannah. Introduction to Meta‐Analysis: Wiley. 2009. 325-30

[68] Drummond. Methods for the Economic Evaluation of Health Care Programmes. 4. ed. Oxford: Oxford University Press; 2015. p. 464.

[69] Bugge C, Sæther EM, Brustugun OT, Kristiansen IS (2021). Societal cost of cancer in Norway -Results of taking a broader cost perspective. Health Policy..

