# Supplementary material for: Evidence on bringing specialised care to the primary level—effects on the Quadruple Aim and cost-effectiveness: a systematic review
Source: BMC Health Serv Res. 2024 Jan 2;24:2. doi: 10.1186/s12913-023-10159-6 (PMC10763279; doi:10.1186/s12913-023-10159-6)
Supplement: Supplementary file 1 — Additional file 1. Search Protocol for the systematic review. [file 12913_2023_10159_MOESM1_ESM.docx]

**Additional File 1**

Name: Additional file 1_Search_Protocol_050222,

Format: word-document (docx),

Title: Search Protocol for the systematic review

COULD SPECIALIZED CARE BE BROUGHT TO THE PRIMARY LEVEL COST-EFFECTIVELY?,

Description: The file contains the search protocol for the used databases.

**Search Protocol for the systematic review**

**COULD SPECIALIZED CARE BE BROUGHT TO THE PRIMARY LEVEL COST-EFFECTIVELY?**

**5.2.2022**

**Pubmed**

Filters:

NOT Meta-analysis, Review, Systematic Review

Language English

**((**

1. Primary Care

"primary health care"[tw] OR "primary care"[tw] OR "primary healthcare"[tw] OR "Primary Health Care"[Mesh] OR "general practice*"[tw] OR "general practitioner*"[tw] OR "family practice*"[tw] OR "community care"[tw] OR "community health"[tw] OR "primary health"[tw] OR "physician practice*"[tw]

AND

1. Secondary care

"Allergists"[Mesh] OR allergology[tw] OR "Cardiologists"[Mesh] OR cardiology[tw] OR "Dermatologists"[Mesh] OR dermatology[tw] OR "Endocrinologists"[Mesh] OR endocrinology[tw] OR "Gastroenterologists"[Mesh] OR gastroenterology[tw] OR "Nephrologists"[Mesh] OR nephrology[tw] OR "Neurologists"[Mesh] OR "Neurology"[tw] OR "Oncologists"[Mesh] OR oncology[tw] OR "Ophthalmologists"[Mesh] OR ophthalmology[tw] OR "Otolaryngologists"[Mesh] OR otolaryngology[tw] OR "Pediatricians"[Mesh] OR "Physiatrists"[Mesh] OR "Pulmonologists"[Mesh] OR pulmonology[tw] OR "Rheumatologists"[Mesh] OR rheumatology[tw] OR "Surgeons"[Mesh] OR "Urologists"[Mesh] OR urology[tw] OR Allergist*[tw] OR Cardiologist*[tw] OR Dermatologist*[tw] OR Endocrinologist*[tw] OR Gastroenterologist*[tw] OR Nephrologist*[tw] OR Neurologist*[tw] OR Oncologist*[tw] OR Ophthalmologist*[tw] OR Otolaryngologist*[tw] OR Pediatrician*[tw] OR Physiatrist*[tw] OR Pulmonologist*[tw] OR Rheumatologist*[tw] OR Surgeon*[tw] OR Urologist*[tw] OR specialist*[tw] OR "secondary care"[tw] OR "secondary health care"[tw] OR "Secondary Care"[Mesh] OR “secondary health”[tw] OR consultant*[tw] OR "vertical integrat*"[Text Word] OR "vertically integrat*"[Text Word]

AND

1. Integration

"Delivery of Health Care, Integrated"[Mesh] OR Embedded[tw] OR "outreach service*"[tw] OR "outreach clinic*"[tw] OR out-of-hospital[tw] OR integrat*[tw] OR substitut*[tw] OR visiting[tw] OR multidisc*[tw] OR "outpatient service*"[tw] OR relocation[tw] OR collabor*[tw]

)

OR

"specialist outreach*"[tw] OR "Outreach specialist*"[tw]

)

AND

1. Quality

quality[tw] OR "Quality of Health Care"[Mesh] OR Outcome*[tw] OR effect*[tw] OR satisf*[tw] OR "Patient Satisfaction"[Mesh] OR wait*[tw] OR effici*[tw] OR "Efficiency, Organizational"[Mesh] OR “unnecessary referral*”[tw] OR "Time Factors"[Mesh] OR “throughput time*”[tw] OR “timely care”[tw] OR “triple aim”[tw] OR “quadruple aim”

AND

1. Cost

"Costs and Cost Analysis"[Mesh] OR cost*[tw] OR expenditure*[tw] OR expense*[tw] OR budget*[tw] OR econom*[tw] OR financ*[tw] OR price*[tw] OR affordable[tw] OR referr*[tw]

((((("Primary Health Care"[Text Word] OR "primary care"[Text Word] OR "primary healthcare"[Text Word] OR "Primary Health Care"[MeSH Terms] OR "general practice*"[Text Word] OR "general practitioner*"[Text Word] OR "family practice*"[Text Word] OR "community care"[Text Word] OR "community health"[Text Word] OR "primary health"[Text Word] OR "physician practice*"[Text Word]) AND ("Allergists"[MeSH Terms] OR "allergology"[Text Word] OR "Cardiologists"[MeSH Terms] OR "cardiology"[Text Word] OR "Dermatologists"[MeSH Terms] OR "dermatology"[Text Word] OR "Endocrinologists"[MeSH Terms] OR "endocrinology"[Text Word] OR "Gastroenterologists"[MeSH Terms] OR "gastroenterology"[Text Word] OR "Nephrologists"[MeSH Terms] OR "nephrology"[Text Word] OR "Neurologists"[MeSH Terms] OR "Neurology"[Text Word] OR "Oncologists"[MeSH Terms] OR "oncology"[Text Word] OR "Ophthalmologists"[MeSH Terms] OR "ophthalmology"[Text Word] OR "Otolaryngologists"[MeSH Terms] OR "otolaryngology"[Text Word] OR "Pediatricians"[MeSH Terms] OR "Physiatrists"[MeSH Terms] OR "Pulmonologists"[MeSH Terms] OR "pulmonology"[Text Word] OR "Rheumatologists"[MeSH Terms] OR "rheumatology"[Text Word] OR "Surgeons"[MeSH Terms] OR "Urologists"[MeSH Terms] OR "urology"[Text Word] OR "allergist*"[Text Word] OR "cardiologist*"[Text Word] OR "dermatologist*"[Text Word] OR "endocrinologist*"[Text Word] OR "gastroenterologist*"[Text Word] OR "nephrologist*"[Text Word] OR "neurologist*"[Text Word] OR "oncologist*"[Text Word] OR "ophthalmologist*"[Text Word] OR "otolaryngologist*"[Text Word] OR "pediatrician*"[Text Word] OR "physiatrist*"[Text Word] OR "pulmonologist*"[Text Word] OR "rheumatologist*"[Text Word] OR "surgeon*"[Text Word] OR "urologist*"[Text Word] OR "specialist*"[Text Word] OR "Secondary Care"[Text Word] OR "secondary health care"[Text Word] OR "Secondary Care"[MeSH Terms] OR "secondary health"[Text Word] OR "consultant*"[Text Word] OR "vertical integrat*"[Text Word] OR "vertically integrat*"[Text Word]) AND ("delivery of health care, integrated"[MeSH Terms] OR "Embedded"[Text Word] OR "outreach service*"[Text Word] OR "outreach clinic*"[Text Word] OR "out-of-hospital"[Text Word] OR "integrat*"[Text Word] OR "substitut*"[Text Word] OR "visiting"[Text Word] OR "multidisc*"[Text Word] OR "outpatient service*"[Text Word] OR "relocation"[Text Word] OR "collabor*"[Text Word])) OR ("specialist outreach*"[Text Word] OR "outreach specialist*"[Text Word])) AND ("Costs and Cost Analysis"[MeSH Terms] OR "cost*"[Text Word] OR "expenditure*"[Text Word] OR "expense*"[Text Word] OR "budget*"[Text Word] OR "econom*"[Text Word] OR "financ*"[Text Word] OR "price*"[Text Word] OR "affordable"[Text Word] OR "referr*"[Text Word]) AND ("quality"[Text Word] OR "Quality of Health Care"[MeSH Terms] OR "outcome*"[Text Word] OR "effect*"[Text Word] OR "satisf*"[Text Word] OR "Patient Satisfaction"[MeSH Terms] OR "wait*"[Text Word] OR "effici*"[Text Word] OR "efficiency, organizational"[MeSH Terms] OR "unnecessary referral*"[Text Word] OR "Time Factors"[MeSH Terms] OR "throughput time*"[Text Word] OR "timely care"[Text Word] OR "triple aim"[Text Word] OR "quadruple aim"[Text Word])) NOT (((("Primary Health Care"[Text Word] OR "primary care"[Text Word] OR "primary healthcare"[Text Word] OR "Primary Health Care"[MeSH Terms] OR "general practice*"[Text Word] OR "general practitioner*"[Text Word] OR "family practice*"[Text Word] OR "community care"[Text Word] OR "community health"[Text Word] OR "primary health"[Text Word] OR "physician practice*"[Text Word]) AND ("Allergists"[MeSH Terms] OR "allergology"[Text Word] OR "Cardiologists"[MeSH Terms] OR "cardiology"[Text Word] OR "Dermatologists"[MeSH Terms] OR "dermatology"[Text Word] OR "Endocrinologists"[MeSH Terms] OR "endocrinology"[Text Word] OR "Gastroenterologists"[MeSH Terms] OR "gastroenterology"[Text Word] OR "Nephrologists"[MeSH Terms] OR "nephrology"[Text Word] OR "Neurologists"[MeSH Terms] OR "Neurology"[Text Word] OR "Oncologists"[MeSH Terms] OR "oncology"[Text Word] OR "Ophthalmologists"[MeSH Terms] OR "ophthalmology"[Text Word] OR "Otolaryngologists"[MeSH Terms] OR "otolaryngology"[Text Word] OR "Pediatricians"[MeSH Terms] OR "Physiatrists"[MeSH Terms] OR "Pulmonologists"[MeSH Terms] OR "pulmonology"[Text Word] OR "Rheumatologists"[MeSH Terms] OR "rheumatology"[Text Word] OR "Surgeons"[MeSH Terms] OR "Urologists"[MeSH Terms] OR "urology"[Text Word] OR "allergist*"[Text Word] OR "cardiologist*"[Text Word] OR "dermatologist*"[Text Word] OR "endocrinologist*"[Text Word] OR "gastroenterologist*"[Text Word] OR "nephrologist*"[Text Word] OR "neurologist*"[Text Word] OR "oncologist*"[Text Word] OR "ophthalmologist*"[Text Word] OR "otolaryngologist*"[Text Word] OR "pediatrician*"[Text Word] OR "physiatrist*"[Text Word] OR "pulmonologist*"[Text Word] OR "rheumatologist*"[Text Word] OR "surgeon*"[Text Word] OR "urologist*"[Text Word] OR "specialist*"[Text Word] OR "Secondary Care"[Text Word] OR "secondary health care"[Text Word] OR "Secondary Care"[MeSH Terms] OR "secondary health"[Text Word] OR "consultant*"[Text Word] OR "vertical integrat*"[Text Word] OR "vertically integrat*"[Text Word]) AND ("delivery of health care, integrated"[MeSH Terms] OR "Embedded"[Text Word] OR "outreach service*"[Text Word] OR "outreach clinic*"[Text Word] OR "out-of-hospital"[Text Word] OR "integrat*"[Text Word] OR "substitut*"[Text Word] OR "visiting"[Text Word] OR "multidisc*"[Text Word] OR "outpatient service*"[Text Word] OR "relocation"[Text Word] OR "collabor*"[Text Word])) OR ("specialist outreach*"[Text Word] OR "outreach specialist*"[Text Word])) AND ("Costs and Cost Analysis"[MeSH Terms] OR "cost*"[Text Word] OR "expenditure*"[Text Word] OR "expense*"[Text Word] OR "budget*"[Text Word] OR "econom*"[Text Word] OR "financ*"[Text Word] OR "price*"[Text Word] OR "affordable"[Text Word] OR "referr*"[Text Word]) AND ("quality"[Text Word] OR "Quality of Health Care"[MeSH Terms] OR "outcome*"[Text Word] OR "effect*"[Text Word] OR "satisf*"[Text Word] OR "Patient Satisfaction"[MeSH Terms] OR "wait*"[Text Word] OR "effici*"[Text Word] OR "efficiency, organizational"[MeSH Terms] OR "unnecessary referral*"[Text Word] OR "Time Factors"[MeSH Terms] OR "throughput time*"[Text Word] OR "timely care"[Text Word] OR "triple aim"[Text Word] OR "quadruple aim"[Text Word]) AND ("meta analysis"[Filter] OR "review"[Filter] OR "systematic review"[Filter]))) AND (1992/1/1:3000/12/12[pdat])

**Scopus**

1. "primary health care" OR "primary care" OR "primary healthcare" OR "general practice*" OR "general practitioner*" OR "family practice*" OR "community care" OR "community health" OR "primary health" OR "physician practice*"
2. Allergist* OR allergology OR Cardiologist* OR cardiology OR Dermatologist* OR dermatology OR Endocrinologist* OR endocrinology OR Gastroenterologist* OR gastroenterology OR Nephrologist* OR nephrology OR Neurologist* OR Neurology OR Oncologist* OR oncology OR Ophthalmologist* OR ophthalmology OR Otolaryngologist* OR otolaryngology OR Pediatrician* OR Physiatrist* OR Pulmonologist* OR pulmonology OR Rheumatologist* OR rheumatology OR Surgeon* OR Urologist* OR urology OR specialist* OR "secondary care" OR "secondary health care" OR "secondary health" OR consultant* OR "vertical* integrat*"
3. Embedded OR "outreach service*" OR "outreach clinic*" OR out-of-hospital OR integrat* OR substitut* OR visiting OR multidisc* OR "outpatient service*" OR relocation OR collabor*
4. (specialist* W/2 outreach*)
5. quality OR Outcome* OR effect* OR satisf* OR wait* OR effici* OR "unnecessary referral*" OR "Time Factor*" OR "throughput time*" OR "timely care" OR "triple aim" OR “quadruple aim”
6. cost* OR expenditure* OR expense* OR budget* OR econom* OR financ* OR price* OR affordable OR referr*

(TITLE-ABS-KEY("primary health care" OR "primary care" OR "primary healthcare" OR "general practice*" OR "general practitioner*" OR "family practice*" OR "community care" OR "community health" OR "primary health" OR "physician practice*")) AND (TITLE-ABS-KEY(Allergist* OR allergology OR Cardiologist* OR cardiology OR Dermatologist* OR dermatology OR Endocrinologist* OR endocrinology OR Gastroenterologist* OR gastroenterology OR Nephrologist* OR nephrology OR Neurologist* OR Neurology OR Oncologist* OR oncology OR Ophthalmologist* OR ophthalmology OR Otolaryngologist* OR otolaryngology OR Pediatrician* OR Physiatrist* OR Pulmonologist* OR pulmonology OR Rheumatologist* OR rheumatology OR Surgeon* OR Urologist* OR urology OR specialist* OR "secondary care" OR "secondary health care" OR "secondary health" OR consultant* OR "vertical integrat*" OR "vertically integrat*")) AND ((TITLE-ABS-KEY(Embedded OR "outreach service*" OR "outreach clinic*" OR out-of-hospital OR integrat* OR substitut* OR visiting OR multidisc* OR "outpatient service*" OR relocation OR collabor*)) OR (TITLE-ABS-KEY("specialist outreach*" OR "Outreach specialist*"))) AND (TITLE-ABS-KEY(quality OR Outcome* OR effect* OR satisf* OR wait* OR effici* OR "unnecessary referral*" OR "Time Factor*" OR "throughput time*" OR "timely care" OR "triple aim" OR “quadruple aim”)) AND (TITLE-ABS-KEY(cost* OR expenditure* OR expense* OR budget* OR econom* OR financ* OR price* OR affordable OR referr*)) AND PUBYEAR > 1991 AND ( EXCLUDE ( DOCTYPE,"re" ) ) AND ( LIMIT-TO ( LANGUAGE,"English" ))

**Ebsco: CINAHL with Full Text, Academic Search Ultimate**

**((**

1. "primary health care" OR "primary care" OR "primary healthcare" OR "general practice*" OR "general practitioner*" OR "family practice*" OR "community care" OR "community health" OR "primary health" OR "physician practice*"

AND

1. Allergist* OR allergology OR Cardiologist* OR cardiology OR Dermatologist* OR dermatology OR Endocrinologist* OR endocrinology OR Gastroenterologist* OR gastroenterology OR Nephrologist* OR nephrology OR Neurologist* OR Neurology OR Oncologist* OR oncology OR Ophthalmologist* OR ophthalmology OR Otolaryngologist* OR otolaryngology OR Pediatrician* OR Physiatrist* OR Pulmonologist* OR pulmonology OR Rheumatologist* OR rheumatology OR Surgeon* OR Urologist* OR urology OR specialist* OR "secondary care" OR "secondary health care" OR “secondary health” OR consultant* OR "vertical integrat*” OR "vertically integrat*"

AND

1. Embedded OR "outreach service*" OR "outreach clinic*" OR out-of-hospital OR integrat* OR substitut* OR visiting OR multidisc* OR "outpatient service*" OR relocation OR collabor*

)

OR

1. (specialist* N2 outreach*))

AND

1. quality OR Outcome* OR effect* OR satisf* OR wait* OR effici* OR "unnecessary referral*" OR "Time Factor*" OR "throughput time*" OR "timely care" OR "triple aim" OR “quadruple aim”

AND

1. cost* OR expenditure* OR expense* OR budget* OR econom* OR financ* OR price* OR affordable OR referr*

(((("primary health care" OR "primary care" OR "primary healthcare" OR "general practice*" OR "general practitioner*" OR "family practice*" OR "community care" OR "community health" OR "primary health" OR "physician practice*") AND (Allergist* OR allergology OR Cardiologist* OR cardiology OR Dermatologist* OR dermatology OR Endocrinologist* OR endocrinology OR Gastroenterologist* OR gastroenterology OR Nephrologist* OR nephrology OR Neurologist* OR Neurology OR Oncologist* OR oncology OR Ophthalmologist* OR ophthalmology OR Otolaryngologist* OR otolaryngology OR Pediatrician* OR Physiatrist* OR Pulmonologist* OR pulmonology OR Rheumatologist* OR rheumatology OR Surgeon* OR Urologist* OR urology OR specialist* OR "secondary care" OR "secondary health care" OR “secondary health” OR consultant* OR "vertical integrat*” OR "vertically integrat*") AND (Embedded OR "outreach service*" OR "outreach clinic*" OR out-of-hospital OR integrat* OR substitut* OR visiting OR multidisc* OR "outpatient service*" OR relocation OR collabor*)) OR (specialist* N2 outreach*) ) AND (quality OR Outcome* OR effect* OR satisf* OR wait* OR effici* OR "unnecessary referral*" OR "Time Factor*" OR "throughput time*" OR "timely care" OR "triple aim" OR "quadruple aim") AND (cost* OR expenditure* OR expense* OR budget* OR econom* OR financ* OR price* OR affordable OR referr*))

| Expanders - Apply equivalent subjects  Search modes - Boolean/Phrase  Limiters - Language: English; Published Date: 19920101-;  Publication Type: Academic Journal, Conference Papers, Country Report, Database, Government Documents, Primary Source Document, Report; Clinical Trial, Journal Article, Research  Document Type: Clinical trial, Conference paper, Journal article, Report, Research, Science experiment;  English Language; Exclude MEDLINE records; |  |
| --- | --- |
