# Supplementary material for: Evidence on bringing specialised care to the primary level—effects on the Quadruple Aim and cost-effectiveness: a systematic review
Source: BMC Health Serv Res. 2024 Jan 2;24:2. doi: 10.1186/s12913-023-10159-6 (PMC10763279; doi:10.1186/s12913-023-10159-6)

**Additional File 3**

Name: Additional file 3

Format: word-document (docx),

Title: Additional file 3

Description: Figure S1 presents the simultaneous directions of effects on cost and quality following the specialist in primary care intervention. It visualises the comparison of quality against the different aspects of costs presented in Tables 4, 5 and 6 in the review. The first column above every category on horizontal axis represents the case in which all the costs and quality outcomes are taken into account (Table 4).The second column presents the case where monetary cost and cost drivers for the commissioner and all the quality outcomes are is taken into account (Table 5). The third column presents the case where only monetary cost for the commissioner against the quality is taken into account (Table 6).

Figure S1. Simultaneous effect directions on cost and quality following the specialist in Primary care intervention.


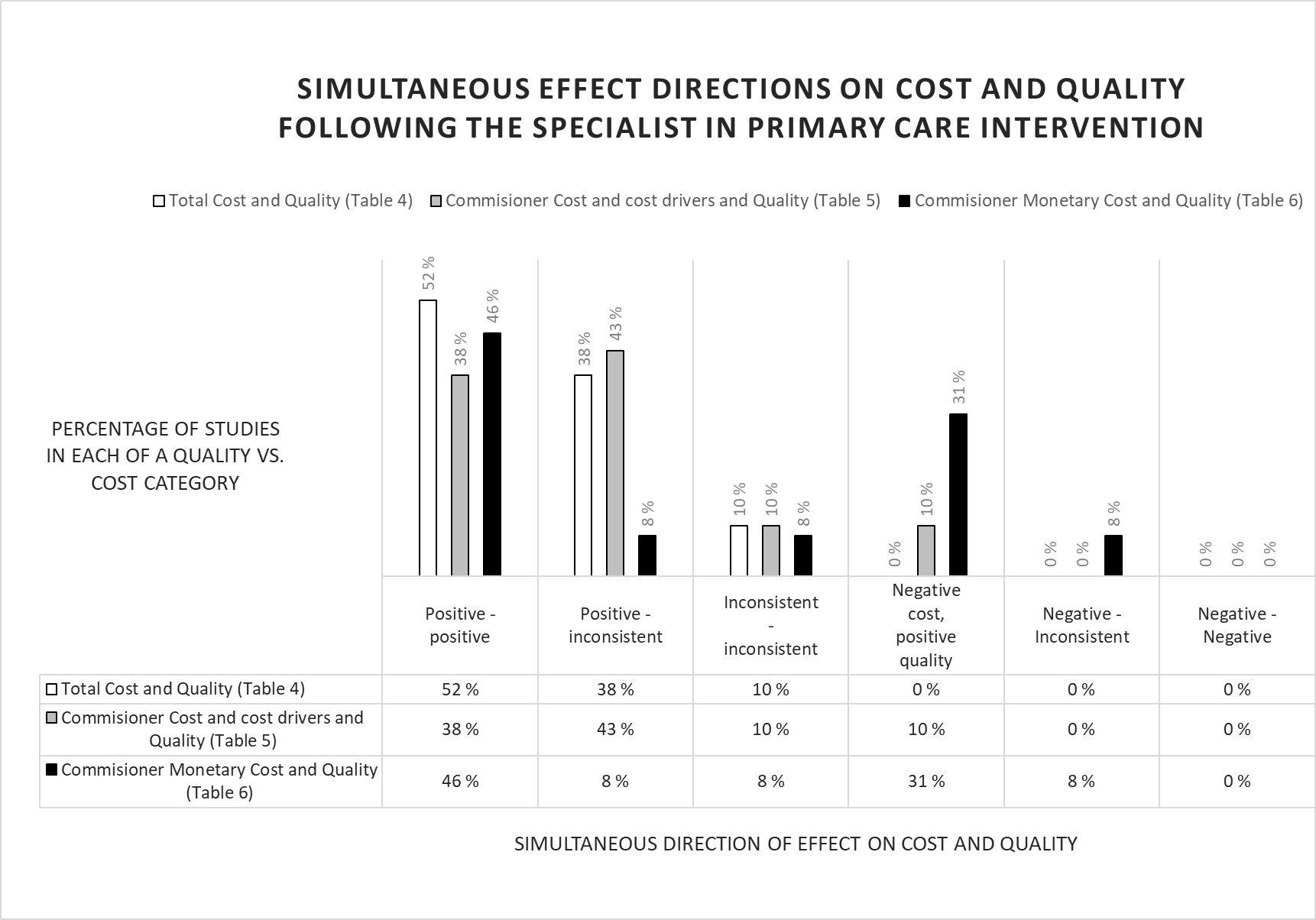

Supplement: Supplementary file 3 — Additional file 3: Figure S1. presents the simultaneous directions of effects on cost and quality following the specialist in primary care intervention. It visualises the comparison of quality against the different aspects of costs presented in Tables 4, 5 and 6 in the review. The first column above every category on horizontal axis represents the case in which all the costs and quality outcomes are taken into account (Table 4).The second column presents the case where monetary cost and cost drivers for the commissioner and all the quality outcomes are is taken into account (Table 5). The third column presents the case where only monetary cost for the commissioner against the quality is taken into account (Table 6). Figure S1. Simultaneous effect directions on cost and quality following the specialist in Primary care intervention. [file 12913_2023_10159_MOESM3_ESM.docx]
