# Supplementary material for: Evidence on bringing specialised care to the primary level—effects on the Quadruple Aim and cost-effectiveness: a systematic review
Source: BMC Health Serv Res. 2024 Jan 2;24:2. doi: 10.1186/s12913-023-10159-6 (PMC10763279; doi:10.1186/s12913-023-10159-6)
Supplement: Supplementary file 4 — Additional file 4: Table S1. includes Clinical outcomes and patient-reported outcome measures (PROMs) and Table S2 includes detailed costs and cost drivers. [file 12913_2023_10159_MOESM4_ESM.docx]

**Additional File 4**

Name: Additional file 4 021122_rev 200823

Format: word-document (docx),

Title: Additional file 4

Description: Table S1 includes Clinical outcomes and patient-reported outcome measures (PROMs) and Table S2 includes detailed costs and cost drivers.

Table S1. Clinical outcomes and patient-reported outcome measures (PROMs)

| **Author (year)** | **Patients Clinical Outcomes** | **PROM** |
| --- | --- | --- |
| Arslan et al. (2021) | Not measured | Not measured |
| Black et al. (1997)  Gosden et al. (1997) | Not measured | Dermatology:  HSQ-12: significantly greater improvement in health status DLQI decline in impairment in the control group, but not among intervention patients in the 3 months follow-up. Orthopaedics:  HSQ-12: no significant difference in the health status at the 3-month follow-up. |
| Bowling et al. (1997) | Not measured | RAND and SF-36 (current HSQ-12) No significant differences in health status or impact on the quality of life between the groups. |
| Bowling et al. (2001)  Bond et al. (2000) | Not measured | HSQ-12.1: Outreach patients had slightly but significantly improved health status change scores in the follow up on the health perceptions and pain dimensions.  The other continuous parameters seemed to improve as well but p>0.05. |
| Dankner et al. (2007) | Not measured | Not measured |
| Davis et al. (2021) | Lipids in 12 months and other parameters in 4 month follow-up: HbA1c reduced 13 mmol/l (baseline 79) in the intervention group LDLs reduced 0.3 mmol/l, Total cholesterol 0.4 mmol/l, and Triglycerides 0.2 mmol/l.  HDLs remained stable. Systolic blood pressure reduced 4 and diastolic 3 mmHg (baseline 132/79) Waist circumference reduced 1 cm.  Medication:  sign increase in the use of GLP-1 and insulin (47% to 74%) between first and last visit. No hypoglycaemia was reported.  All above changes were statistically significant. BMI and smoking: no significant change. | Not measured |
| Donald et al. (2021) | Clinical outcomes were non-inferior to those with gold-standard hospital-based outpatient services without harm. | Not measured |
| Elrashidi et al. (2017) | Not measured | Not measured |
| Gillam et al. (1995) | Not measured | Not measured |
| Gillett et. al. (2016) | Respiratory exacerbations reduced by 67.6%. Non-elective respiratory GP practice visits reduced by 78.5% and planned visits reduced by 28.6%. Respiratory emergency department (ED)/hospital admissions reduced from 3 to 0. 23.6% received a change in diagnosis after review. SABA prescribed decreased 33.3%, ICS-containing inhalers prescribed increased by 23.3%. | Not measured |
| Gruen et al. (2001) | Not measured | Not measured |
| Gruen et al. (2006) | Not measured | Not measured |
| Hiscock et al. (2020) | 24/142 (17%) of children with bronchiolitis or bronchitis were prescribed unnecessary steroids or antibiotics by their GP. This fell to 10/114 9% after the intervention had started.  Unnecessary prescribing of acid suppression medication fell in infants under 1 year of age with irritability, crying, reflux or unsettled behaviour from 14/48 (29%) to 5/53(9%)  Fewer unnecessary tests (not specified more accurately). | Not measured |
| Hu et al. (2021) | 18-month follow-up: **Hba1c** under control in 80% vs. 79% in the precision group vs. regular treatment Delay for 80% of the group to reach the set goals: 3 months in precision management group vs. 5.5 months for the regular management group.  **Blood pressure** under control 91% vs. 89% in precision group vs. regular treatment Delay for 90% of the group to reach under-control rate: 3 months in precision group vs. 4.5 months in regular group.  Probability to achieve an under-control status was higher in the precision management group, and patients achieved control faster than patients in the regular management group.  2014-2016 Hypertension: the precision management group had over 40% higher under-control rate than that of the regular management group;  Diabetes: the precision management group had over 30% higher under-control rate than their counterpart group. | Not measured |
| Leiba et al. (2002) | Not measured | Not measured |
| McLeod H et al. (2015) | Not measured | Not measured |
| Montgomery-Taylor et al. (2016) | Not measured | Not measured |
| Philpot et al. (2021) | Not measured | Not measured |
| Quanjel et al. (2019) | Not measured | EQ-5D-5L, EQ-VAS, SF-12:  No significant differences between the groups over time. |
| Sibbald et al. (2008) | Not measured. | Not measured |
| Young et al. (2017) | No cases met criteria for an adverse outcome. | Not measured. |

PROM = patient reported outcome measurement, HSQ-12 = Health Status Questionnaire-12, DLQI = Dermatology Life Quality Index, SF-36 = 36-Item Short Form Health Survey, HbA1c= haemoglobin A1c, LDL = low-density lipoprotein, HDL = high-density lipoprotein, BMI = body-mass index, SABA = short-acting beta-agonist, ICS = inhaled corticosteroids, EQ-5D = EuroQol-5 Dimension - an instrument for measuring quality of life, EQ-VAS = EuroQol-visual analogue scale

Table S2. Costs and cost drivers.

| **Author (year)** | **MONETARY COST** 1) For the commissioner 2) For the patient | **COST DRIVERS Resources related outcomes: from the record (Quantitative)** 1) Non-attendance rates 2) Referrals to the hospital/specialist /follow-up visit on a specialist 3) Referral to laboratory or imaging or other investigation 4) Expenditure of health services 5) Expenses for the patient (travel time and distance, absence from the work etc.) 6) Other | **Notes** |
| --- | --- | --- | --- |
| Arslan et al. (2021) | Not measured | 2) 16% of the patients from the intermediate care sent to the orthopaedist vs. 43% from the regular GP care. In total, there was no significant difference in referrals to the orthopaedist pre- and post-implementation.   4) Interviews: Lower healthcare costs due to better selection of patients to secondary care and less healthcare consumption. | 4) Interviews: Lower healthcare costs due to better selection of patients to secondary care and less healthcare consumption. 5) Interviews: Lower out-of-pocket costs and shorter travel distances for a patient |
| Black et al. (1997) | Dermatology outreach clinics had significantly lower health service costs/patient including treatment and overhead costs (average difference -£20.14, 95% CI: -£38.68 to -£1.61). For Orthopedics the total Health service cost is not reported, Gosden: In orthopaedic: no significant result.   The cost of treating an additional patient (marginal cost) higher in both specialties (average difference of £4.17, 95% CI: £3.24 to £5.09 for dermatology; average difference of £9.59, 95% CI: £4.98 to £14.19 for orthopaedics).  Gosden:  Breakdown of the health service costs per patient per clinic below Staff: Dermatology outreach £6.27, outpatient £3.62. Orthopaedic outreach £9.60, outpatient £6.09. Staff travel and opportunity cost: Dermatology outreach £1.52, outpatient not applicable (NA), Orthopaedic outreach £9.60, outpatient NA. Marginal cost: Dermatology outreach £7.79, outpatient clinic below Staff: Dermatology outreach £6.27, outpatient £3.62. Orthopaedic outreach £9.60, outpatient £6.09. Staff travel and opportunity cost: Dermatology outreach £1.52, outpatient not applicable (NA), Orthopaedic outreach £9.60, outpatient NA. Marginal cost: Dermatology outreach £7.79, outpatient £3.62, Orthopaedic outreach £15.68, outpatient £6.09. (p <0.001)  Overhead costs: Dermatology outreach £2.78, outpatient £8.69, Orthopaedic no significant results Prescription: Dermatology outreach £6.86, outpatient £11.69, Orthopaedic no significant results. Test and investigation: Dermatology outreach £5.75, outpatient £5.23, Orthopaedic no significant results Procedure costs: Dermatology outreach £20.60, outpatient £34.69, Orthopaedic no significant results  -> Dermatology total cost lower/patient, but marginal cost higher in Outreach in both specialities.  2) Large but not significant differences in favour of outreach in the patient travel, time and total patient costs (reported in £) | 1. No statistically significant difference (lower at dermatology outreach: 11% vs. 20%. Higher at orthopaedic outreach: 9% vs. 3%) Outcome of the consultation in orthopaedics 2) Recall for follow-up consultation significantly less in the outreach Placed on waiting list for surgery significantly more often in the outreach 3) Number of tests (other than blood and urine) significantly (p = 0.013) less in the outreach clinic within dermatology 5) Travel times less at dermatology outreach 20 min vs. 40 min. Orthopaedics: no significant difference | Casemix data suggests: Dermatology outreach and outpatients differed in the type and severity of their condition, while for Orthopaedics the distribution of treatment costs highly skewed because significantly more outreach patients were put on waiting lists for high-cost surgical procedures.   Overhead costs: Variations in the content and quality of information provided by practices and hospitals. “Results suggest that like was not compared with like.” |
| Bowling et al. (1997) | Not measured/reported. Cost analysis ongoing. | 2) Less follow-ups after the visit from the outreach 37 vs. 50%  89% reduction in hospital referrals in 6 weeks after starting the outreach (data from the manager) Rheumatology 37% referred for therapy vs. 14% outpatients.  3) Outreach patients less likely to have any tests required 30% vs. 57% of the outpatients  *Prescribed/suggested treatment: 76% outreach vs. 67% outpatient  5) Less in outreach: 62% vs. 20% outpatients travelled <3 miles to the clinic and 60% vs. 28% outpatient travelled <10minutes.  Time off work: 50% of outreach patients vs. 24% of outpatients took max 1 hour off work, 25% of outreach vs. 32% (12) of outpatients 2 hours and 25% outreach vs. 44% outpatients took >2 hours. | Journey distances and times to the clinic significantly shorter in the outreach clinic, as well as the time off the work. |
| Bowling et al. (2001)  Bond et al. (2000) | 1) Total NHS treatment costs per patient higher in the outreach £149.59 vs. £106.79. Of these, treatment £135.21 vs. £96.26 and staffing £14.38 vs. £10.53. 2) Total cost to the patient lower in the outreach £3.96 vs. £8.40. Of these, travel £0.82 vs. £2.08, care for dependants £0.05 vs. £0.11 and opportunity cost (time) £3.9 vs. £6.21.  1) Total NHS treatment costs per patient higher in the outreach 163,73 vs. 109,20£  The opportunity cost of staff travel times and travel costs higher in the outreach 15,52 vs 11,07£  Staffing cost per patient higher in the outreach 13,80 vs. 11,07£  NHS overhead costs are not given bacause figures were not standardized  2) Total cost to the patient lower in the outreach 4,55 vs. 9,97£. | 1) Non-attendance rates lower at outreach 10% vs. 16% 2) Less follow-ups or referrals to therapy, surgery or other services, that is, more outreach patient completely discharged after the visit: significant for follow-up patients 30% vs. 21% but not for new patients  1) Non-attendance rates lower at the outreach clinic 10% vs. 16%  2) less follow-ups, that is more outreach patient completely discharged after the visit 36% vs. 27% | Costs included specialists’ travel costs (outreach only), NHS staffing costs, overhead costs, patients’ treatment and prescription costs, patients’ personal costs (e.g. travel, costs of carers, child-minders), and time costs (travel and waiting times)  2) Interview: 1/3 reduction in referrals to the specialist (managers note),  46% of GPs and 33% of specialist reported a decline in a number of referrals - 13% GPs and 6% specialists reported instead increase. |
| Dankner et al. (2007) | Not measured | In the intervention group: 2) 31% less referrals to the tertiary care, 65% less to ED. No sign difference in follow-up 3) 31% less referrals to the laboratory compared to control clinics. Imaging no difference. 5) No significant difference in sick leave days |  |
| Davis et al. (2021) | Not measured | 1) Non-attendance <10% in the intervention (in the literature 39% in usual outpatient care) 2) Referrals to the hospital reduced remarkably after the outreach and started to elevate again after the funding end of the outreach. |  |
| Donald et al. (2021) | 1) The incremental cost saving per patient 365A$ in the intervention (i.e. total cost per patient course of treatment 365A$ less in the intervention): 2622A$ vs. 2987A$ in the outpatient hospital care.  Cost per visit lower in the intervention: 403A$ vs. 622A$ in usual care. Costs included: Endocrinologist, GPs, nurses, administration staff, overhead costs. Excluded consumables, radiology, pathology, and medicines because their use was assumed to be similar.  Excluded also consequences of the better Hba1c control in the intervention. | Not measured |  |
| Elrashidi et al. (2017) | Not measured | 3) Less EMG (OR 0.64) and brain MRI (OR 0.60) in the intervention. No difference in ODDs of EEG, CT head, MRI spine or MRA head and neck. 4) Fewer subsequent neurology visits in the intervention group (OR 0.62).  No difference in total subsequent outpatient visits, emergency department visits or hospitalization. |  |
| Gillam et al. (1995) | 1) Total cost/ophthalmologist session does not differ: outreach £394.34 vs. hospital £397.57.  Total cost per patient in the intervention 3 times that of the hospital: £48.09 vs. £15.71.   Costs including staffing, travel, medication/disposables, overheads and depreciation in equipment. | 5) Distances travelled: 1.2% of the intervention patients travelled over 5 miles compared 22% of the hospital controls.  Travel times: 73.9% of the intervention patients travelled <10 minutes and 0% over 50 minutes, compared to 18.7% and 12% of the hospital patients.  8.3% outreach vs. 33.3% outpatients paying to travel and 26.1% in the outreach vs. 45.3% outpatients reported requiring an escort for the visit |  |
| Gillett et. Al. (2016) | 1) Cost of the exacerbation avoided £142.89. The incremental cost-effectiveness of the intervention versus no intervention is £142.89 per exacerbation avoided. Respiratory-related costs per patient over a 9-month period decrease from £458.11 to £226.25.  Cost of the intervention £296.82/patient. | 2) 7.3% were referred on secondary care for further investigations (no pre-intervention data available) 4) Non-elective respiratory GP visits -78.5%, elective respiratory GP visits -28.6%, Respiratory Practice nurse PN visits -47.7% (in all p <0.01).  In total primary care visits reduced by 53% (p <0.01).  Admissions to emergency reduced from 3 to 0 pre- vs. post intervention in 9 month follow up (p <0.05). 67.3% required primary care appointment (no pre-intervention data available). |  |
| Gruen et al. (2001) | 1) Cost of a consultation less in outreach clinic $277 vs. $357 in the regional hospital and $450 at the outpatient clinic at Royal Darwin Hospital | Not measured |  |
| Gruen et al. (2006) | Not measured | 1-4) Gruen (2006) Outreach was associated with a reduced rate of procedures that needed hospital admission (adjusted hazard ratio 0.67, 0.43-.03).  When regular outreach clinics were available the proportion of problems that were electively referred for specialist procedures and the proportion of those referred that eventually had the procedure were no different to when no regular outreach clinics were available (83% [74/89] vs 80%) |  |
| Hiscosk et al. (2021) | Cost estimate:   1. 1) A$ 260 822 above usual care per 624 children in 12 months which is extra costs A$ 417.98 / children. 2. 2) A$ 153216 below usual care per 624 in 12 months which is savings A$ 245.54 / children | 3 ) Trend towards fewer referrals to:  emergency departments : before 26(19%) vs. after the intervention 15(12%) and  Private Peadiatrician 46(24%) vs. 25(20%) (not significant).  Referrals to outpatient clinics remained the same until increased towards end of the pilot as the paediatrician left the clinics 42(31%) vs.59(47%)  5) Is estimated in the calculation of monetary costs. |  |
| Hu et al. (2021) | 1) Total direct treatment expenses in the precision management group from 2014 to 2016 were for the:  Hypertensive patients 238 CNY lower than that in the regular management group and 381 CNY lower than in the without management group and for Diabetic patients 704 CNY lower than that in the regular management group and 1117 CNY lower than that in the without management group. | Not measured | 2016 the treatment costs of the precision management group for hypertension were around 300 CNY higher than costs for the regular management group, and around 800 CNY higher than costs for the without management group. However, after the reduction of possible endogeneity based on PSM+DID analyses. Intervention had better cost-saving effects in practise. |
| Leiba et al. (2002) | 1) In the intervention clinic no significant change in total monthly cost of medical services: $186.8 vs. $177.1 pre- vs. post-intervention. In the control clinic no significant change in monthly cost of medical services: $111.6 vs. $120.9 after intervention and no significant increase in the loss of working days (1849 to 2025). | 2) Out-of-clinic referrals to the military regional centre reduced from 1449 to 421, at the same time also referrals to the hospital and emergency outpatient reduced significantly. 4) No increased use in overall medical services. Not significant reduction in visits to primary care. No significant change in either in referrals or any visits. 5) Loss of work days reduced significantly from 2891 to 1938 after the intervention.  In the control clinic Loss of work days increased, but not significantly, from 1849 to 2025. | While referrals and self-referrals to the outreach increased. |
| McLeod H et al. (2015) | One of the two outreach clinics was efficient (measured by the ratio of income to staff related cost) compared with most hospital outpatient clinics. The exact ratio and sum are not announced. The intervention clinic with short clinic duration, shorter new referral appointment slots and larger proportion of time booked with appointments, was more cost-efficient. | 1) Non-attendance rate did not significantly differ between the intervention clinics (new patients 15.8%, follow-up patients 18.1%) and the hospitals (new patients 14.2%, follow up patients 15.8%).  Compared with the least deprived quartile of the population, children living in the most-deprived two quartiles were 1.8 times more likely to not attend to the appointment.  5) Accessibility – distance to the intervention clinic 2.9 km and 3.2 km, to the hospital 5.6 km and 11.9 km. | Using national data on NHS staff costs19 and NHS tariff prices for general paediatric attendances.  Income to staff-related cost – ratio varies between the clinics and no significant announced in the article. |
| Montgomery-Taylor et al. (2016) |  | 1) Non-attendance rates lower in the outreach (<5%) compared with the local hospital (>15%). 2) New referrals to general paediatric outpatients reduced in the intervention clinics an average 69% ( 81%, 63% and 64%). In the control clinic, referrals increased 23%. To that new referrals to subspecialty paediatric outpatients decreased 19%, 31% and 15% while those increased in the control GP 5%. 4) 17% reduction in the admissions to the hospital, 22% decrease in A&E attenders. 39% of new patient hospital appointments were avoided altogether and a further 42% of appointments were shifted from hospital to GP practice.   Paediatric outpatient appointments were remarkably reduced post-intervention and compared to non-intervention practices. No statistical significance was calculated. | 19% decrease in subspecialty referrals, 75% completely discharged after the appointment without any follow up 17% sent to another health professional 6% recommended specialist investigation. 2% follow-up visit at outreach vs. approximately 50% in outpatients.  17% reduction in the admissions to the hospital, 22% decrease in A&E attenders. 39% of new patient hospital appointments were avoided altogether and a further 42% of appointments were shifted from hospital to GP practice. Paediatric outpatient appointments were remarkably reduced post-intervention and compared to non-intervention practices. No statistical significance was calculated.   Multidisciplinary meetings (MDT) In 59% of cases (91/154) the referring community-based professional was given advice that enabled the child to be managed in primary care, 21% (32/154) were directed  to the paediatric outreach clinic for an appointment the following month and, in the remaining 20% (31/154) of cases, the professional who had brought the case to the MDT meeting was advised to refer the patient directly to specific named health professionals such as hospital specialty paediatricians, community dieticians, physiotherapists or child and adolescent mental health services (CAMHS). |
| Philpot et al. (2021) | Not measured | 3) No differences in diagnostic test utilization pre- and post-intervention. 4) Intervention patients had significant decreased likelihood for outpatient GI visits for all condition groups except GI neoplasms: diarrhoea OR = 0.3 GERD/esophagitis OR = 0.4, GI bleed/anaemia OR = 0.4, IBS/abdominal pain OR = 0.4 (GI neoplasms OR = 0.6, 95% CI 0.2–1.4; p = 0.20).  GERD/esophagitis patients had increased likelihood of more primary care visits, OR = 1.5 (p = 0.02). No significant differences in visits to other outpatient specialty practices, visits to the emergency department, or number of hospital discharges. | Visit utilization: fewer visits to the standard GI referral practice without increased use of health services, except increased likelihood of visits with primary care. |
| Quanjel et al. (2019) | 1) The healthcare costs per patient were significantly lower (p <0.001) and increased less in the intervention group in the baseline, 3, 6, and 9 months follow-up:  the cost was  in the baseline 107.72 euros less after 3 months 289.93 euros less after 6 months 367.97 euros less after 9 months 471.75 euros less. compared to the control group. | Not measured | Age and gender were not confounding variables in the cost calculations. Cost of cardiological healthcare per patient (incl. diagnostic tests, consultations, surgeries, and services in the outreach and hospital, NO primary care costs) |
| Sibbald et al. (2008) | Estimated cost per patient less in the intervention: Dermatology -£79.61 (-52%) Orthopaedics -£122.43 (-42%) gynecology -£39.88 (-14%) General surgery -£113.31 (-20%) - No consultant ENT - No consultant Urology -£133.17 (-46%) - No consultant | Not measured | New services dealt with less complex conditions and undercut the price tariff applied to hospitals thus providing a cost saving to commissioners |
| Umesh et al. (2011) Umesh et al. (2015) | 1. 4000£ for the intervention of 6 months (excl. 15h hours of work of the unspecified actor).   Reseacher has approximated savings of 13.50£ in the 2011 article and 13£ in the 2015 article, per every 1£ spent to reduce Hba1c by the intervention described, includes several assumptions and does not include labour cost.   1. Not measured. | Not measured | In was not opened wat was included in the cost calculation of the intervention. The labour cost was excluded. To that there was many assumptions and the number of the porilation was small. |
| Young et al. (2017) | Not measured | 2) The number of referrals to tertiary neurology declined by 64% after the intervention, and the total number of face‐to‐face visits per month declined by 25%. 4) Outreach resulted in avoidance of 78 face‐to‐face tertiary neurology consultations, 39 brain magnetic resonance imaging, 50 electromyograms, and 53 other advanced imaging studies. Earlier curbside consultation may have prevented unnecessary testing or face‐to‐face tertiary neurology consultations in 40 (22%) patients. Earlier face‐to‐face consultation may have avoided expensive testing in 31 (17%) patients. | Pure costs in currency not calculated. |

GP = general practitioner, NHS = National Health Service, ED = emergency department, EMG = electromyogram, EEG = electroencephalography, CT= computerized tomography, MRI = magnetic resonance imaging, MRA= magnetic resonance angiography, PN = practical nurse, CNY = Chinese yuan renminbi, GERD = gastroesophageal reflux disease, IBS = irritable bowel syndrome, GI = gastrointestinal, ENT = Ear nose and throat, A$=Australian dollar, OR=odds ratio
