# Supplementary material for: Evidence on bringing specialised care to the primary level—effects on the Quadruple Aim and cost-effectiveness: a systematic review
Source: BMC Health Serv Res. 2024 Jan 2;24:2. doi: 10.1186/s12913-023-10159-6 (PMC10763279; doi:10.1186/s12913-023-10159-6)
Supplement: Supplementary file 5 — Additional file 5: Table S3. includes GRADE appraisal of the outcomes. [file 12913_2023_10159_MOESM5_ESM.docx]

**Additional File 5**

Name: Additional file 5

Format: word-document (docx),

Title: Additional file 5

Description: Table S3 includes GRADE appraisal of the outcomes

**Taable S1. The result of the GRADE appraisal.**

**Author(s):** Lovén Maria, Pitkänen Laura

**Question:** Specialist compared to GP in primary care to improve Quadruple Aim and cost-efficiency

**Setting:** Hospital specialist in primary care

**Bibliography:**

| **Certainty assessment** | | | | | | | **№ of patients** | | **Effect** | | **Certainty** | **Importance** |
| --- | --- | --- | --- | --- | --- | --- | --- | --- | --- | --- | --- | --- |
| **№ of studies** | **Study design** | **Risk of bias** | **Inconsistency** | **Indirectness** | **Imprecision** | **Other considerations** | **specialist** | **GP** | **Relative (95% CI)** | **Absolute (95% CI)** |  |  |
| **Cost-effectiveness (assessed with: ICER)** | | | | | | | | | | | | |
| 2 | observational studies | not serious | not serious | not serious | serious | none | 55^1,2,b^ | 55 | - | see comment | ⨁⨁⨁◯ Moderate | IMPORTANT |
| **Population health (assessed with: Clinical outcomes and standardized life quality questionnaires)** | | | | | | | | | | | | |
| 10 | observational studies^1,2,3,4,5,6,7,8,9,10,c^ | serious^d^ | not serious | serious^e^ | not serious | none | 20919 | 189990 | - | see comment | ⨁⨁◯◯ Low | IMPORTANT |
| **Patient experience (assessed with: PREM and Process outcomes mostly waitimg times)** | | | | | | | | | | | | |
| 18 | observational studies^1,2,4,5,7,8,9,10,11,12,13,14,15,16,17,18,19,20,f^ | not serious^g^ | not serious | not serious | serious^h^ | none | 8920 | 5412 | - | see comment | ⨁⨁⨁◯ Moderate | IMPORTANT |
| **Professional satisfaction** | | | | | | | | | | | | |
| 10 | observational studies^5,8,9,10,11,12,15,16,17,21^ | not serious | not serious | serious^i^ | not serious^j^ | none | 7920 | 2199 | - | see comment | ⨁⨁⨁◯ Moderate | IMPORTANT |
| **Costs including cost drivers** | | | | | | | | | | | | |
| 20 | observational studies^1,2,3,4,5,6,7,8,9,10,11,12,13,14,15,17,19,20,21,22,23^ | serious^k^ | serious^l^ | serious^m^ | serious^n^ | none | 37844 | 198330 | - | see comment | ⨁◯◯◯ Very low | IMPORTANT |

**CI:** confidence interval

#### Explanations

a. No exact number of patient partisipants announced in the economical evaluation of Donald.

b. In the article of donald no exact number of patients announced.

c.

d. Serious risk of bias on half of the studies, no difference in the weight of the studies.

e. Differencies in outcome measures, surrogate outcomes used.

f. Patient experience measured in many different study types: quasi-experimental, cohort, case-control, case series.

g. 5 of the studies seriously or moderately biased, though the patient experience measures mostly proper questionnaires. Some based on interviews.

h. Includes also interviews and unstandardized questionnaires without numerical assessment.

i. Professional often reporting surrogate outcomes like their opinion of the effect of the intervention to patient care.

j. In majority of the studies interviews used and no numerical values available, the questions vary between the studies, and some do not exactly measure satisfaction.

k. There was bias due to confounding in some of the studies which affect also the cost calculation.

l. Costs for the commissioner and the patient in different direction in part of the studies, which is though explainable.

m. Also surrogate measures included.

n. Approximations without confidence intervels in part of the studies. Values of the cost drivers mostly unclear.

#### References

1.Gillett, K., Lippiett, K., Astles, C., Longstaff, J., Orlando, R., Lin, S. X., Powell, A., Roberts, C., Chauhan, A. J., Thomas, M., Wilkinson, T. M.. Managing complex respiratory patients in the community: an evaluation of a pilot integrated respiratory care service. BMJ Open Respir Res; 2016.

2.Donald, M., Jackson, C. L., Byrnes, J., Vaikuntam, B. P., Russell, A. W., Hollingworth, S. A.. Community-based integrated care versus hospital outpatient care for managing patients with complex type 2 diabetes: costing analysis. Aust Health Rev; Feb 2021.

3.Young, N. P., Elrashidi, M. Y., Crane, S. J., Ebbert, J. O.. Pilot of integrated, colocated neurology in a primary care medical home. J Eval Clin Pract; Jun 2017.

4.Quanjel, Tessa C. C., Spreeuwenberg, Marieke D., Struijs, Jeroen N., Baan, Caroline A., Ruwaard, Dirk. Substituting hospital-based outpatient cardiology care: The impact on quality, health and costs. PLOS ONE; 2019.

5.Hiscock, H. Strenghtening care for children: pilot of an integrated general practitioner-paediatrician model of primary care in victoria, australia. Australian Health Review; 2020.

6.Hu, H., Liang, H., Wang, H.. Longitudinal study of the earliest pilot of tiered healthcare system reforms in China: Will the new type of chronic disease management be effective?. Soc Sci Med; Sep 2021.

7.Davis, T. M. E., Drinkwater, J. J., Fegan, P. G., Chikkaveerappa, K., Sillars, B., Davis, W. A.. Community-based management of complex type 2 diabetes: adaptation of an integrated model of care in a general practice setting. Intern Med J; Jan 2021.

8.Bowling, A., Stramer, K., Dickinson, E., Windsor, J., Bond, M.. Evaluation of specialists&#x27; outreach clinics in general practice in England: process and acceptability to patients, specialists, and general practitioners. Journal of Epidemiology &amp; Community Health; 1997.

9.Bowling, A., Bond, M.. A national evaluation of specialists&#x27; clinics in primary care settings. Br J Gen Pract; Apr 2001.

10.Black, M., Leese, B., Gosden, T., Mead, N.. Specialist outreach clinics in general practice: what do they offer?. Br J Gen Pract; Sep 1997.

11.Sibbald, B., Pickard, S., McLeod, H., Reeves, D., Mead, N., Gemmell, I., Coast, J., Roland, M., Leese, B.. Moving specialist care into the community: an initial evaluation. J Health Serv Res Policy; Oct 2008.

12.Montgomery-Taylor, S., Watson, M., Klaber, R.. Child Health General Practice Hubs: a service evaluation. Arch Dis Child; Apr 2016.

13.Leiba, Adi, Martonovits, Giora, Magnezi, Rachel, Goldberg, Avishay, Carroll, Judith, Benedek, Paul, Ohana, Nissim, Leiba, Ronit, Bar-Dayan, Yaron. Evaluation of a specialist outreach clinic in a primary healthcare setting: the effect of easy access to specialists. Clinician in Management; 2002.

14.McLeod, H., Heath, G., Cameron, E., Debelle, G., Cummins, C.. Introducing consultant outpatient clinics to community settings to improve access to paediatrics: an observational impact study. BMJ Qual Saf; Jun 2015.

15.Gruen, R. L., Bailie, R. S., d'Abbs, P. H., O'Rourke, I. C., O'Brien, M. M., Verma, N.. Improving access to specialist care for remote Aboriginal communities: evaluation of a specialist outreach service. Med J Aust; May 21 2001.

16.Gosden, T., Black, M., Mead, N., Leese, B.. The efficiency of specialist outreach clinics in general practice: is further evaluation needed?. J Health Serv Res Policy; Jul 1997.

17.Gillam, S. J., Ball, M., Prasad, M., Dunne, H., Cohen, S., Vafidis, G.. Investigation of benefits and costs of an ophthalmic outreach clinic in general practice. Br J Gen Pract; Dec 1995.

18. Bond M. Evaluation of outreach clinics held by specialists in general practice in England. Journal of Epidemiology & Community Health. 2000;54(2):149-56.

19. Dankner R, Rieck J, Bentacur AG, Bar Dayan Y, Shahar A. Civilian doctors in military clinics--outsourcing for better medicine. Mil Med. 2007;172(1):75-8.

20.Gruen, Russel,L., Bailie, Ross, Wang , Zhiqiang,, Heardd , Sam, O'Rourke , Ian. Specialist outreach to isolated and disadvantaged communities: a population-based study. Lancet; 2006.

21.Arslan, I. G., Voorbrood, V. M. I., Stitzinger, S. A. G., van de Kerkhove, M. P., Rozendaal, R. M., van Middelkoop, M., Bindels, P. J. E., Bierma-Zeinstra, S. M. A., Schiphof, D.. Evaluation of intermediate care for knee and hip osteoarthritis: a mixed-methods study. BMC Fam Pract; Jun 24 2021.

22.Philpot, L. M., Ramar, P., Sanchez, W., Ebbert, J. O., Loftus, C. G.. Effect of Integrated Gastroenterology Specialists in a Primary Care Setting: a Retrospective Cohort Study. J Gen Intern Med; May 2021.

23.Elrashidi, M. Y., Philpot, L. M., Young, N. P., Ramar, P., Swanson, K. M., McKie, P. M., Crane, S. J., Ebbert, J. O.. Effect of integrated community neurology on utilization, diagnostic testing, and access. Neurol Clin Pract; Aug 2017.
