# Supplementary material for: Evidence on bringing specialised care to the primary level—effects on the Quadruple Aim and cost-effectiveness: a systematic review
Source: BMC Health Serv Res. 2024 Jan 2;24:2. doi: 10.1186/s12913-023-10159-6 (PMC10763279; doi:10.1186/s12913-023-10159-6)
Supplement: Supplementary file 6 — Additional file 6: Table S4. presents the ED plot of secondary outcomes sorted by Risk of Bias. [file 12913_2023_10159_MOESM6_ESM.docx]

**Additional File 6**

Name: Additional file 6

Format: word-document (docx),

Title: Additional file 6

Description: Table S4 presents the ED plot of secondary outcomes sorted by Risk of Bias.

Table S4. Effect Direction plot of secondary outcomes
